# Supplementary material for: Repurposing Dantrolene for Long-Term Combination Therapy to Potentiate Antisense-Mediated DMD Exon Skipping in the mdx Mouse
Source: Mol Ther Nucleic Acids. 2018 Feb 13;11:180–91. doi: 10.1016/j.omtn.2018.02.002 (PMC5992346; doi:10.1016/j.omtn.2018.02.002)
Supplement: Document S1. Figures S1–S8 [file mmc1.pdf]

OMTN, Volume *11*

Supplemental Information

Repurposing Dantrolene for Long-Term Combination Therapy to Potentiate Antisense-Mediated *DMD* Exon Skipping in the mdx Mouse

Derek W. Wang, Ekaterina I. Mokhonova, Genevieve C. Kendall, Diana Becerra, Yalda B. Naeini, Rita M. Cantor, Melissa J. Spencer, Stanley F. Nelson, and M. Carrie Miceli

Supplementary Material

Repurposing Dantrolene for Long-term Combination Therapy to Potentiate Antisense-Mediated *DMD* Exon-Skipping in the mdx mouse

Supplementary Figure 1. Oral Dantrolene synergizes with systemically delivered e23AON to promote exon 23 skipping and restoration dystrophin expression after 3 weeks treatment.

(a) Effect of Dantrolene on e23AON-induced skipped dystrophin protein as measured by quantitative immunofluorescence of individual quadriceps and diaphragms. Mice were dosed systemically with once weekly e23AON 50 mg/kg. One cross-section per muscle per animal was evaluated for dystrophin expression, and data are presented a percent of C57 control levels of dystrophin. Error bars represent one standard deviation from the mean. n = 4 – 5. ‡ Dantrolene was dosed at 30-70mg/kg/day. (*P≤0.05 compared to e23kAON only control by two-tailed t-test.)

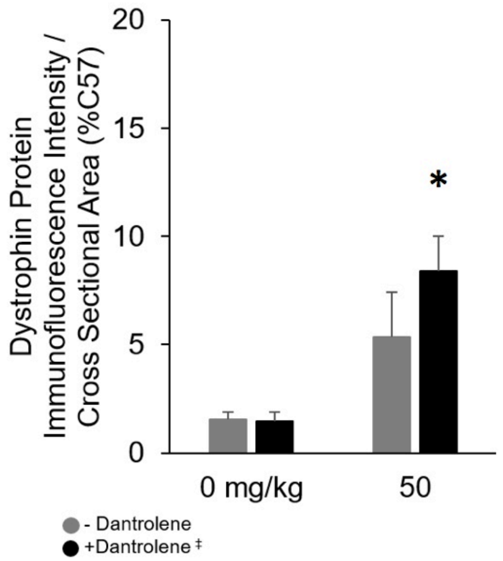

Supplementary Figure 2. Dantrolene was detected at physiologically relevant levels in the serum of mice fed with dantrolene chow.

(a) Serum was isolated from all mice after 26 weeks of treatment. Dantrolene was detected by ELISA. Each sample was run in duplicate. Variability reflects differences between individual mice, not duplicates.To account for 1:5 dilution of sample necessary for dantrolene ELISA, values were multiplied by 5 to calculate final serum levels. n = 60. (b) A standard curve was included on each plate to ensure linearity and all experimentally determined points were within the limits of the dantrolene standard curve.

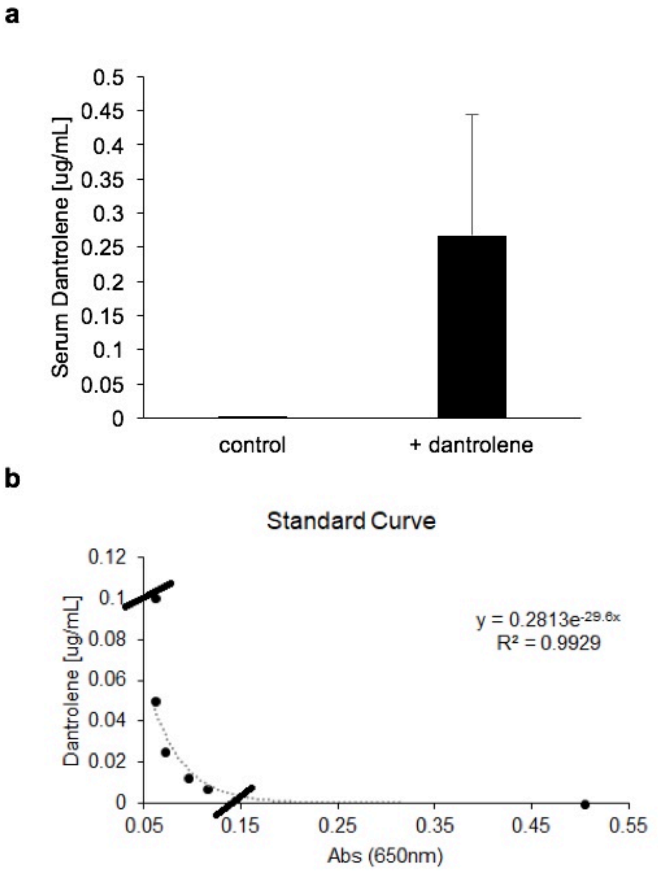

Supplementary Figure 3. Results of Friedman's two-way nonparametric ANOVA on the effect of dantrolene on e23AON treatment for exon 23 skipped / (skipped + unskipped) transcript as measured by ddPCR.

For this analysis, quantitative ddPCR readout of skipped/(skipped+unskipped) for exon-skipping in each mouse was ranked. Once ranked, the findings for each muscle, considered separately, were first fitted for PMO, followed by dantrolene, and the possibility of their interaction.

Friedman's Two-way Nonparametric ANOVA

The ANOVA Procedure

Dependent Variable: RPCR_Q Rank for Variable PCR_Q

Source	DF	Sum of Squares	Mean Square	F Value	Pr > F
Model	7	125825.9029	17975.1290	57.16	<.0001
Error	117	36794.5971	314.4837		
Corrected Total	124	162620.5000			

R-Square	Coeff Var	Root MSE	RPCR_Q Mean
0.773739	28.14871	17.73369	63.00000

Source	DF	Anova SS	Mean Square	F Value	Pr > F
PMO	3	121276.2635	40425.4212	128.55	<.0001
Dantrolene	1	2051.5705	2051.5705	6.52	0.0119
PMO*Dantrolene	3	2498.0689	832.6896	2.65	0.0522

Dependent Variable: RPCRD Rank for Variable PCRD

Source	DF	Sum of Squares	Mean Square	F Value	Pr > F
Model	7	203225.7682	29032.2526	71.56	<.0001
Error	138	55990.2318	405.7263		
Corrected Total	145	259216.0000			

R-Square	Coeff Var	Root MSE	RPCRD Mean
0.784002	27.40496	20.14265	73.50000

Source	DF	Anova SS	Mean Square	F Value	Pr > F
PMO	3	198329.0385	66109.6795	162.94	<.0001
Dantrolene	1	4756.2345	4756.2345	11.72	0.0008
PMO*Dantrolene	3	140.4953	46.8318	0.12	0.9509

Supplementary Figure 4. Results of Friedman's two-way nonparametric ANOVA for the addition of dantrolene to e23AON treatment for dystrophin immunofluorescence quantitation and dystrophin positive fibers.

P-values are from Friedman's test, a two-way nonparametric ANOVA, and meta-analysis of the results across muscles. For this analysis, quantitative dystrophin immunofluorescence quantitation and dystrophin positive fibers.readout in each mouse was ranked.

Friedman's Two-way Nonparametric ANOVA

The ANOVA Procedure

Dependent Variable: RC57_Q   Rank for Variable C57_Q

Source	DF	Sum of Squares	Mean Square	F Value	Pr > F
Model	7	229837.8073	32833.9725	69.48	<.0001
Error	145	68523.1927	472.5737		
Corrected Total	152	298361.0000			

R-Square	Coeff Var	Root MSE	RC57_Q Mean
0.770335	28.23216	21.73876	77.00000

Source	DF	Anova SS	Mean Square	F Value	Pr > F
PMO	3	222163.5627	74054.5209	156.70	<.0001
Dantrolene	1	4256.7113	4256.7113	9.01	0.0032
PMO*Dantrolene	3	3417.5333	1139.1778	2.41	0.0693

Dependent Variable: RC57D   Rank for Variable C57D

Source	DF	Sum of Squares	Mean Square	F Value	Pr > F
Model	7	300546.6800	42935.2400	188.79	<.0001
Error	151	34341.8200	227.4293		
Corrected Total	158	334888.5000			

R-Square	Coeff Var	Root MSE	RC57D Mean
0.897453	18.85095	15.08076	80.00000

Source	DF	Anova SS	Mean Square	F Value	Pr > F
PMO	3	299112.7209	99704.2403	438.40	<.0001
Dantrolene	1	677.9429	677.9429	2.98	0.0863
PMO*Dantrolene	3	756.0161	252.0054	1.11	0.3478

Dependent Variable: RPostiveQ   Rank for Variable PostiveQ

Source	DF	Sum of Squares	Mean Square	F Value	Pr > F
Model	7	246586.4318	35226.6331	81.65	<.0001
Error	147	63418.5682	431.4188		
Corrected Total	154	310005.0000			

R-Square	Coeff Var	Root MSE	RPostiveQ Mean
0.795427	26.62901	20.77062	78.00000

Source	DF	Anova SS	Mean Square	F Value	Pr > F
PMO	3	239888.7374	79962.9125	185.35	<.0001
Dantrolene	1	3234.0959	3234.0959	7.50	0.0069
PMO*Dantrolene	3	3463.5985	1154.5328	2.68	0.0494

Dependent Variable: RPostiveD   Rank for Variable PostiveD

Source	DF	Sum of Squares	Mean Square	F Value	Pr > F
Model	7	316449.4676	45207.0668	185.92	<.0001
Error	154	37446.5324	243.1593		
Corrected Total	161	353896.0000			

R-Square	Coeff Var	Root MSE	RPostiveD Mean
0.894188	19.13321	15.59357	81.50000

Source	DF	Anova SS	Mean Square	F Value	Pr > F
PMO	3	312734.6671	104244.8890	428.71	<.0001
Dantrolene	1	2141.8141	2141.8141	8.81	0.0035
PMO*Dantrolene	3	1572.9864	524.3288	2.16	0.0955

Supplementary Figure 5. Results of Friedman's two-way nonparametric ANOVA on the effect of dantrolene on e23AON treatment for dystrophin protein by western blot.

P-values are from Friedman's test, a two-way nonparametric ANOVA, and meta-analysis of the results across muscles. For this analysis, quantitative readout of dystrophin protein by western blot.in each mouse was ranked.

Friedman's Two-way Nonparametric ANOVA

The ANOVA Procedure

Dependent Variable: RWB_Q Rank for Variable WB_Q

Source	DF	Sum of Squares	Mean Square	F Value	Pr > F
Model	7	135014.2588	19287.7513	42.27	<.0001
Error	124	56582.7412	456.3124		
Corrected Total	131	191597.0000			

R-Square	Coeff Var	Root MSE	RWB_Q Mean
0.704678	32.12251	21.36147	66.50000

Source	DF	Anova SS	Mean Square	F Value	Pr > F
PMO	3	130985.5562	43661.8521	95.68	<.0001
Dantrolene	1	3662.6436	3662.6436	8.03	0.0054
PMO*Dantrolene	3	366.0590	122.0197	0.27	0.8488

Supplementary Figure 6. Combination therapy rescues expression of dystrophin with presence of N-terminal, rod domain, and C-terminal protein sequences.

Dystrophin was detected with antibodies specific to the N-terminal domain (Manex1A), rod domain (MANDYS8), and C-terminal domain (Ab15277) , respectively. ‡"—" defined as 0mg/kg daily dose dantrolene. "+" defined as 30-70mg/kg daily dose dantrolene.

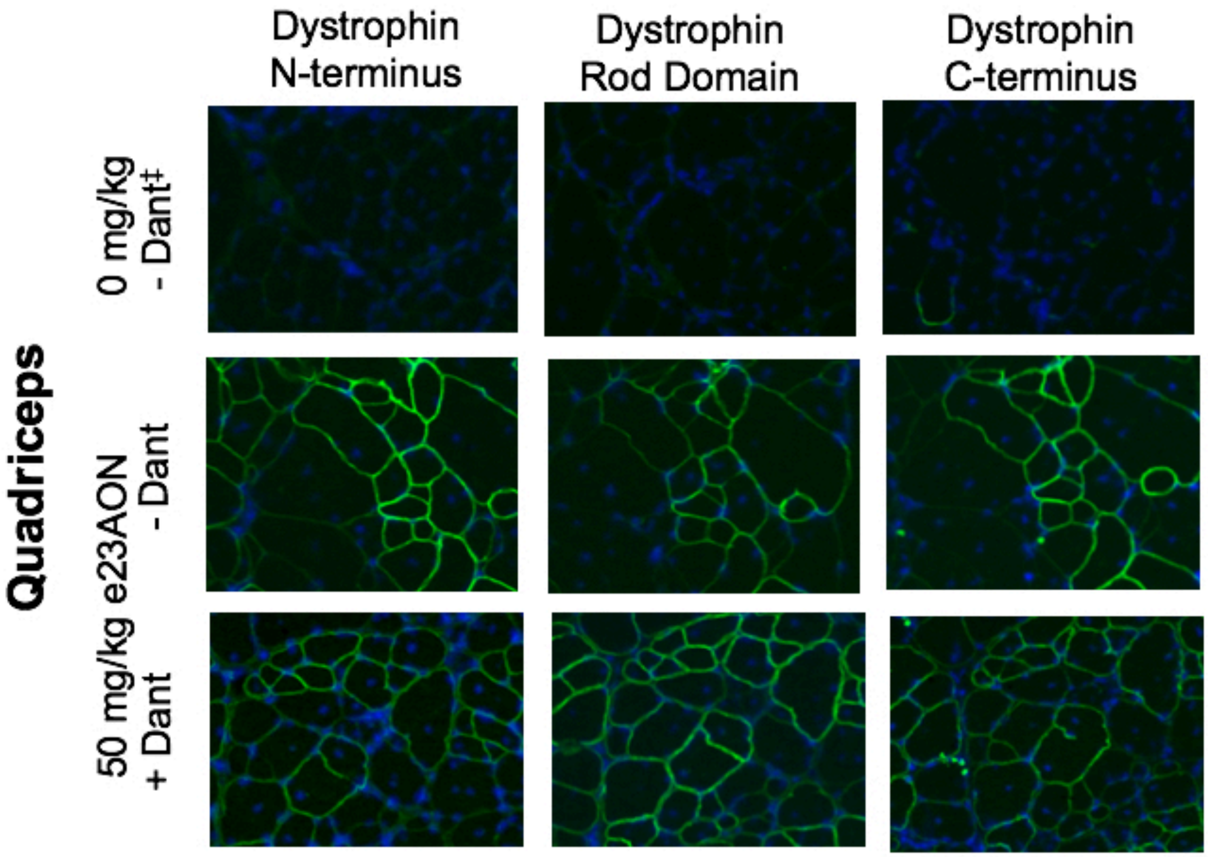

Supplementary Figure 7. Results of Friedman's two-way nonparametric ANOVA on the effect of dantrolene on e23AON treatment for serum CK levels.

P-values are from Friedman's test, a two-way nonparametric ANOVA, and meta-analysis of the results across muscles. For this analysis, quantitative readout of serum CK levels in each mouse was ranked.

Friedman's Two-way Nonparametric ANOVA

The ANOVA Procedure

Dependent Variable: RCK_Serum   Rank for Variable CK_Serum

Source	DF	Sum of Squares	Mean Square	F Value	Pr > F
Model	7	76303.8689	10900.5527	11.38	<.0001
Error	125	119736.6311	957.8930		
Corrected Total	132	196040.5000			

R-Square	Coeff Var	Root MSE	RCK_Serum Mean
0.389225	46.19380	30.94985	67.00000

Source	DF	Anova SS	Mean Square	F Value	Pr > F
PMO	3	30801.59067	10267.19689	10.72	<.0001
Dantrolene	1	39072.39425	39072.39425	40.79	<.0001
PMO*Dantrolene	3	6429.88396	2143.29465	2.24	0.0872

Supplementary Figure 8. Results of Friedman's two-way nonparametric ANOVA on the effect of dantrolene on e23AON treatment for centronucleation and eMHC positive fibers. P-values are from Friedman's test, a two-way nonparametric ANOVA, and meta-analysis of the results across muscles. For this analysis, quantitative readout for centronucleation and eMHC positive fibers in each mouse was ranked.

Friedman's Two-way Nonparametric ANOVA

The ANOVA Procedure

Dependent Variable: RCentroQ Rank for Variable CentroQ

Source	DF	Sum of Squares	Mean Square	F Value	Pr > F
Model	7	1501.416667	214.488095	2.02	0.0769
Error	40	4256.083333	106.402083		
Corrected Total	47	5757.500000			

R-Square	Coeff Var	Root MSE	RCentroQ Mean
0.260776	42.10261	10.31514	24.50000

Source	DF	Anova SS	Mean Square	F Value	Pr > F
PMO	3	993.1666667	331.0555556	3.11	0.0369
Dantrolene	1	379.6875000	379.6875000	3.57	0.0662
PMO*Dantrolene	3	128.5625000	42.8541667	0.40	0.7518

Dependent Variable: RCentroD Rank for Variable CentroD

Source	DF	Sum of Squares	Mean Square	F Value	Pr > F
Model	7	4773.000000	681.857143	7.82	<.0001
Error	40	3489.000000	87.225000		
Corrected Total	47	8262.000000			

R-Square	Coeff Var	Root MSE	RCentroD Mean
0.577705	38.12013	9.339433	24.50000

Source	DF	Anova SS	Mean Square	F Value	Pr > F
PMO	3	3973.500000	1324.500000	15.18	<.0001
Dantrolene	1	546.750000	546.750000	6.27	0.0165
PMO*Dantrolene	3	252.750000	84.250000	0.97	0.4183

Dependent Variable: ReMHCQ Rank for Variable eMHCQ

Source	DF	Sum of Squares	Mean Square	F Value	Pr > F
Model	7	6486.250000	926.607143	13.62	<.0001
Error	40	2722.250000	68.056250		
Corrected Total	47	9208.500000			

R-Square	Coeff Var	Root MSE	ReMHCQ Mean
0.704376	33.67192	8.249621	24.50000

Source	DF	Anova SS	Mean Square	F Value	Pr > F
PMO	3	5587.375000	1862.458333	27.37	<.0001
Dantrolene	1	391.020833	391.020833	5.75	0.0213
PMO*Dantrolene	3	507.854167	169.284722	2.49	0.0743

Dependent Variable: ReMHCD Rank for Variable eMHCD

Source	DF	Sum of Squares	Mean Square	F Value	Pr > F
Model	7	6192.946429	884.706633	17.67	<.0001
Error	38	1902.553571	50.067199		
Corrected Total	45	8095.500000			

R-Square	Coeff Var	Root MSE	ReMHCD Mean
0.764986	30.10986	7.075818	23.50000

Source	DF	Anova SS	Mean Square	F Value	Pr > F
PMO	3	5742.540559	1914.180186	38.23	<.0001
Dantrolene	1	272.695652	272.695652	5.45	0.0250
PMO*Dantrolene	3	177.710217	59.236739	1.18	0.3290
