# Supplementary material for: Repurposing Dantrolene for Long-Term Combination Therapy to Potentiate Antisense-Mediated DMD Exon Skipping in the mdx Mouse
Source: Mol Ther Nucleic Acids. 2018 Feb 13;11:180–91. doi: 10.1016/j.omtn.2018.02.002 (PMC5992346; doi:10.1016/j.omtn.2018.02.002)
Supplement: Document S2. Article plus Supplemental Information [file mmc2.pdf]

Molecular Therapy
Nucleic Acids
Original Article

Repurposing Dantrolene for Long-Term Combination Therapy to Potentiate Antisense-Mediated *DMD* Exon Skipping in the mdx Mouse

Derek W. Wang,[1,2] Ekaterina I. Mokhonova,[1,2,3] Genevieve C. Kendall,[1,4,5] Diana Becerra,[1,3] Yalda B. Naeini,[6] Rita M. Cantor,[4] Melissa J. Spencer,[1,3] Stanley F. Nelson,[1,4,5,6,7] and M. Carrie Miceli[1,2,5,7]

[1]Center for Duchenne Muscular Dystrophy, University of California, Los Angeles, Los Angeles, CA, USA; [2]Department of Microbiology, Immunology, and Molecular Genetics, David Geffen School of Medicine and College of Letters and Sciences, University of California, Los Angeles, Los Angeles, CA, USA; [3]Department of Neurology, David Geffen School of Medicine, University of California, Los Angeles, Los Angeles, CA, USA; [4]Department of Human Genetics, David Geffen School of Medicine, University of California, Los Angeles, Los Angeles, CA, USA; [5]Molecular Biology Institute, University of California, Los Angeles, Los Angeles, CA, USA; [6]Department of Pathology and Laboratory Medicine, University of California, Los Angeles, Los Angeles, CA, USA

Duchenne muscular dystrophy (DMD) is caused by mutations in DMD, resulting in loss of dystrophin, which is essential to muscle health. DMD "exon skipping" uses anti-sense oligonucleotides (AONs) to force specific exon exclusion during mRNA processing to restore reading frame and rescue of partially functional dystrophin protein. Although exon-skipping drugs in humans show promise, levels of rescued dystrophin protein remain suboptimal. We previously identified dantrolene as a skip booster when combined with AON in human DMD cultures and short-term mdx dystrophic mouse studies. Here, we assess the effect of dantrolene/AON combination on DMD exon-23 skipping over long-term mdx treatment under conditions that better approximate potential human dosing. To evaluate the dantrolene/AON combination treatment effect on dystrophin induction, we assayed three AON doses, with and without oral dantrolene, to assess multiple outcomes across different muscles. Meta-analyses of the results of statistical tests from both the quadriceps and diaphragm assessing contributions of dantrolene beyond AON, across all AON treatment groups, provide strong evidence that dantrolene modestly boosts exon skipping and dystrophin rescue while reducing muscle pathology in mdx mice ($p < 0.0087$). These findings support a trial of combination dantrolene/AON to increase exon-skipping efficacy and highlight the value of combinatorial approaches and Food and Drug Administration (FDA) drug re-purposing for discovery of unsuspected therapeutic application and rapid translation.

INTRODUCTION

Duchenne muscular dystrophy (DMD) is the most common lethal genetic disease of childhood, with an incidence that ranges from 10.7 to 27.8 per 100,000 population.[1] DMD is mainly caused by frameshifting multi-exon deletions in the *DMD* gene, resulting in loss of expression of the encoded protein, dystrophin, which is critical to muscle cell health.[2] Dystrophin stabilizes the muscle membrane in the context of contraction by linking the cytoskeleton to the extracellular matrix through N-terminal association with F-actin and C-terminal association with the dystrophin-associated glycoprotein complex (DGC). The DGC spans the membrane and binds laminin a2 within the basal lamina and requires dystrophin for its sarcolemmal localization, stability, and function. Additionally, dystrophin and the DGC contribute to muscle cell signaling and stem cell self-renewal.[3]

Loss of dystrophin and DGC expression results in muscle membrane damage, altered signaling, and defective muscle regeneration. Membrane damage drives increased intracellular Ca^{2+} levels, calpain activation, protein degradation, and cell death.[4] Proper nitric oxide synthase (NOS) localization at the DGC is lost in the absence of dystrophin, resulting in hyper-nitrosylation of the ryanodine receptor 1 (RyR1),[5] which also contributes to Ca^{2+} dysregulation. Inflammation and replacement of muscle with fat and fibrosis further interferes with muscle regeneration and function. Over time, this leads to progressive loss of body-wide skeletal muscle and cardiac function. Boys with DMD typically become dependent on a wheelchair between ages 10 and 13 years and often succumb to respiratory and/or cardiac failure between the ages of 18 and 30 years.

Becker muscular dystrophy (BMD), a milder form of dystrophy, is also caused by mutations in *DMD*.[6,7] However, in most instances, *DMD* mutations in those with BMD preserve the mRNA reading frame and lead to the production of an internally deleted dystrophin protein with partial functionality. BMD patients can range in severity from loss of ambulation at 16 years old to asymptomatic, depending

Received 5 November 2017; accepted 6 February 2018;
https://doi.org/10.1016/j.omtn.2018.02.002.

[7]These authors contributed equally to this work.

Correspondence: M. Carrie Miceli, Department of Microbiology, Immunology, and Molecular Genetics, David Geffen School of Medicine and College of Letters and Sciences, University of California, Los Angeles, Los Angeles, CA, USA.
E-mail: cmiceli@ucla.edu

 © 2018 The Authors.
This is an open access article under the CC BY license (http://creativecommons.org/licenses/by/4.0/).

largely on the stability and functionality of the mutant dystrophin. Of note, several in-frame Becker-causing *DMD* mutations cluster within a hotspot between exons 45 and 55 and encode internally deleted dystrophin proteins with partial functionality responsible for a milder disease course relative to Duchenne.[8] These findings provide the rationale for therapies aimed at reframing mRNA to rescue Becker-like dystrophin proteins with partial functionality.

One therapeutic strategy, termed exon skipping, forces targeted exon exclusion during pre-mRNA splicing to restore the *DMD* reading frame through systemic administration of anti-sense oligo-nucleotides (AONs).[9–11] Reframing by exon skipping rescues the expression of a BMD-like internally deleted dystrophin. AON-guided *DMD* exon skipping has proven effective in rescuing a Becker-like dystrophin protein and improving skeletal muscle function in both mouse and dog DMD models.[12–14]

Exondys51, a morpholino AON designed to restore the *DMD* reading frame by excluding exon 51, was recently granted accelerated approval in the USA and is relevant to 13% of DMD patients.[15–21] In a small multi-year study, 12 boys who were amenable to reframing by skipping exon 51 were administered Exondys51 weekly by intravenous infusion.[17] All patients received 30 mg/kg, except for one who received 50 mg/kg. After 3 years, those treated with Exondys51 were reported to walk 151 m further than predicted by a longitudinal natural history of DMD boys from a cohort well-matched for genotype, age, and functional parameters at study initiation. By year 4, only 20% of Exondys51-treated subjects were reported to have lost ambulation, whereas 80% of the matched historical control lost ambulation over the same time frame.[17–19]

Skeletal muscle biopsies from subjects administered Exondys51 demonstrated a statistically significant induction of dystrophin relative to pre-treatment control biopsies and, ultimately, accelerated approval was granted based on this biomarker.[16,18–21] Levels of induced dystrophin ranged from 0% to 2.47% of normal (as determined by western blot), distributed across an average of 16% of myofibers (as determined by immunofluorescence of dystrophin-positive fibers). Although no lower threshold requirement for dystrophin expression has been firmly established, very low levels have been reported to be associated with some functional benefit in mouse models and human DMD/BMD.[22–24] Some BMD patients have been reported to express as low as 3% of wild-type levels of internally deleted dystrophin.[22,23] Although these patients present with "severe BMD," they are less severe than typical DMD. Western blot analysis has not been performed to allow robust quantitation against a human dystrophin protein standard, limiting clear delineation of the lower limit of dystrophin needed for any degree of functional benefit. Nonetheless, induction of higher levels of dystrophin is predicted to impart greater function.

We previously identified dantrolene as a booster of antisense-mediated exon skipping through an unbiased small molecule screen and validated its efficacy in short-term *mdx* mouse and human DMD myotube culture models.[25] The mdx mouse has a premature termination within exon 23 of *DMD*, leading to loss of dystrophin and muscular dystrophy. Intravenous administration of e23AON promotes exon 23 skipping and rescue of an internally deleted dystrophin protein that is capable of significant functionality and rescue of the DMD-like phenotype.[26,27] We have reported that dantrolene (10 mg/kg), administered twice daily intraperitoneally (i.p.), boosted low-dose (10 mg/kg) AON-directed exon 23 skipping (e23AON), dystrophin protein expression, and strength in the *mdx* mice over the course of short-term experiments (1–3 weeks). Further, DMD patient-derived reprogrammed myotubes bearing an exon 45–50 deletion treated with the dantrolene/e51AON combination induced greater levels of in-frame skipped DMD exon51 mRNA than either drug alone, establishing potential relevance to human DMD treatment.[25]

Here, we assess if dantrolene has an exon skip boosting activity in the *mdx* DMD mouse model in the context of long-term 6-month chronic treatment with weekly intravenous (i.v.) e23AON at a high (300 mg/kg), medium (50 mg/kg), or low (10 mg/kg) dose in combination with dantrolene dosed orally. These conditions are more relevant to potential combination therapy in DMD patients currently prescribed ExonDys51 or who may be exposed to other AON drugs currently in development. We demonstrate that dantrolene boosts e23AON-directed *DMD* mRNA exon skipping, dystrophin protein expression, and rescue of dystrophic pathology in *mdx* mice in the context of long-term chronic treatment, without observed toxicity. These findings provide data in support of the feasibility and efficacy of repurposing dantrolene as a skip-boosting drug for use in combination with exon-skipping AON. Because dantrolene and Exondys51 are both Food and Drug Administration (FDA)-approved drugs with good safety profiles in DMD, combination therapy could be tested in humans.

RESULTS

Orally Administered Dantrolene Enhances Antisense-Mediated Exon Skipping and Dystrophin Protein Rescue in mdx Mice

Previous studies using twice daily i.p. injections identified dantrolene as a booster of DMD exon skipping in short-term treatment using non-clinical grade dantrolene (Sigma). Dantrolene is already clinically available in tablet form for oral administration under the trade name dantrium. Because oral dosing is much more practical both for long-term mouse studies and translation to human DMD, we performed a series of 3-week pilot studies to determine if orally administered dantrium incorporated into chow *ad libitum* boosted dystrophin induction of systemically administered e23AON. We found that orally administered dantrolene administered in combination with 50 mg/kg e23AON boosted dystrophin immunofluorescence intensity per cross-sectional area relative to e23AON treatment alone (Figure S1). Therefore, we relied on oral dantrium incorporated into chow to assess dosing, efficacy, and safety during the course of chronic combination treatment.

A

# of Mice	e23AON (mg/kg)	Dantrolene (mg/kg)
24	0	0
24	0	57±12
24	10	0
24	10	55±6
24	50	0
24	50	54±8
18	300	0
18	300	54±13
**180**		

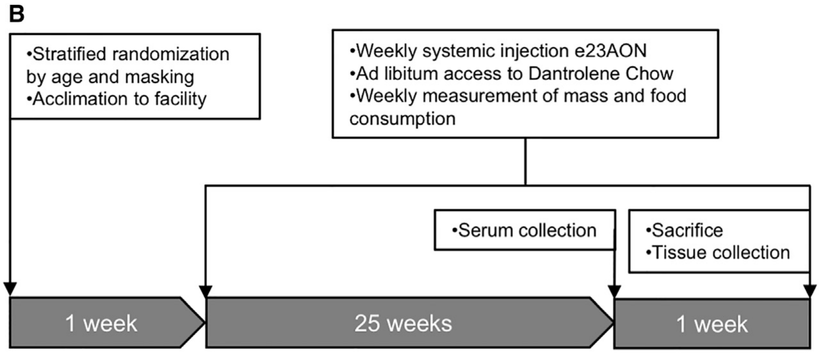

Figure 1. Overview of Long-Term Dosing Experiment

(A) Treatment groups for 6- to 8-week-old mdx mice subjected to 6 months of combination therapy. (B) Protocol outline for long-term dosing experiment.

6-Month Combination Therapy Promotes Increased Exon Skipping and Dystrophin Expression in Skeletal Muscle

We next assessed the efficacy of long-term administration of combination therapy of e23AON and dantrolene in *mdx* mice. We designed a blinded, placebo-controlled, multi-dose, 6-month study, in which *mdx* mice were fed a diet containing 30–70 mg/kg/day dantrolene *ad libitum* in combination with weekly retro-orbital injections of e23AON at either 0, 10, 50, or 300 mg/kg (Figure 1). Experiments were blinded and performed within recently published guidelines.[28] At the beginning of the study, 6- to 8-week-old mice underwent stratified randomization based on age and weight. Mice were acclimated to the facility for 1 week. Then, using a staggered start, cohorts of 9 mice (from random treatment groups) were initiated into the experiment because our workflow is limited to harvest of muscles of 9 mice per day. Mice were weighed weekly and dantrolene chow dose was adjusted to maintain consistent dosing throughout the study. Serum levels of dantrolene were assessed at the end of the treatment period and demonstrated levels of 0.27 ± 0.18 μg/mL were achieved (Figures S2A–S2C).

During the 6-month treatment, mice did not show any change in body weight or feeding habits with dantrolene treatment. Because of the potential for liver and kidney damage upon chronic treatment, we assessed serum levels of γ-glutamyl transferase (GGT), creatinine, bilirubin, and blood urea nitrogen (BUN) 1 week prior to sacrificing animals. We found no significant changes in these measures that were reflective of kidney or liver toxicity (Figure 2) with dantrolene treatment. Although dantrolene treatment did lead to an 18% decrease in serum BUN levels in all treated groups (Figure 2), this decrease is not likely to be biologically significant. H&E staining did not reveal any differences in pathology of the heart, liver, and kidney associated with e23AON and dantrolene dosing (not shown).

After 6 months of administration, levels of *DMD* mRNA exon skipping were determined in the quadriceps and diaphragm using droplet digital PCR (ddPCR) (Figure 3A). The presence or absence of dantrolene skip boosting activity was assessed across multiple e23AON concentrations and quadriceps and diaphragm muscles. Each outcome was tested separately using the same analytic approach to assess whether dantrolene contributes to that outcome beyond e23AON. Because these outcomes are not normally distributed, a nonparametric two-way ANOVA (Friedman's test) was used.[29,30] The p values for each outcome were then combined over the two muscle types using a meta-analysis. To illustrate, for the Friedman test of quantitative ddPCR readouts for *DMD* exon 23 skipping, every mouse was ranked across all AON and dantrolene levels, and a model that includes e23AON levels and the presence or absence of dantrolene as well as the possibility of their interaction was tested for both the quadriceps and diaphragm. Results indicate that e23AON has a highly significant positive treatment effect when considered across all e23AON concentrations combined relative to placebo-treated controls in both the quadriceps and diaphragm analyzed independently ($p < 0.0001$ for each, Figure S3). Further, dantrolene/e23AON combination treatment significantly boosts mRNA exon skipping relative to e23AON treatment alone when all e23AON concentrations tested are considered together ($p = 0.012$ for quadriceps, $p = 0.0008$ for diaphragm; Figures 3B and S3). Meta-analysis of the p values from the quadriceps and diaphragm combined demonstrate a higher level of significance of dantrolene/e23AON treatment on exon skipping ($p = 0.00012$) (Figure 3B).

Dystrophin protein expression in mice treated with combination therapy, both by quantitating the intensity of dystrophin immunofluorescence staining (Figure 4A) and the percentage of dystrophin-positive fibers (Figure 4B) within transverse muscle sections, was also significantly boosted when dantrolene was used in combination with e23AON relative to e23AON alone for dystrophin immunofluorescence intensity in the quadriceps ($p = 0.0032$) and for dystrophin-positive fibers in the quadriceps ($p = 0.0069$) and diaphragms ($p = 0.0035$) (Figures 4A–4D and S4). Statistical significance was not achieved for dystrophin immunofluorescence intensity in the diaphragm when analyzed independently ($p = 0.086$). However, the meta-analysis of p values from the quadriceps and diaphragm strongly supports an effect of dantrolene in combination with

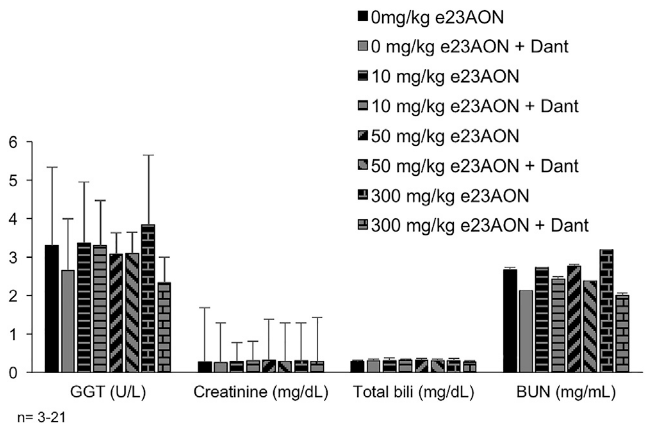

Figure 2. 6-Month Treatment with 10, 50, and 300 mg/kg e23AON with Dantrolene Did Not Adversely Affect Serum Biomarkers and Histopathology

The levels of the following serum enzymes were analyzed: γ-glutamyltransferase (GGT) (U/L), creatinine (mg/dL), total bilirubin (mg/dL), and blood urea nitrogen (BUN) (mg/mL). A significant reduction in BUN levels was observed with the addition of dantrolene in mice receiving 0, 10, 50, and 300 mg/kg e23AON when compared to their e23AON-only counterparts. (*$p \leq 0.05$ compared to e23AON-only control; $^{\#}p \leq 0.01$ compared to e23AON-only control; $^{\dagger}p \leq 0.001$ compared to e23AON-only control. Two-tailed t test) Error bars indicate one SD.

A

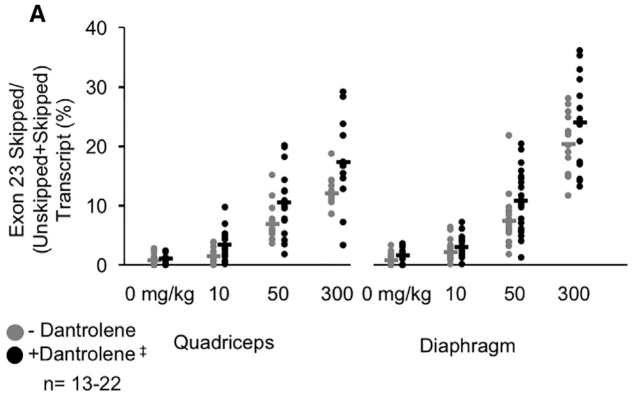

B

		Friedman's Two-way ANOVA by ranks		
Outcome Measure	Relative Fold Change with Dantrolene Treatment	Quadriceps (p-value)	Diaphragm (p-value)	Meta analysis
Exon 23 skipped / (skipped + unskipped) transcript (%)	1.37	0.012	0.0008	0.00012

Figure 3. Dantrolene Boosts e23AON to Promote RNA Exon 23 Skipping

(A) Effect of dantrolene on e23AON induction of *Dmd* exon 23 skipping as determined by the ratio of skipped/(skipped + unskipped) DMD mRNA for the quadriceps and diaphragm. mRNA from each muscle in each treatment group was quantified independently. Each point represents a single mouse; ‡Dantrolene was dosed at 30–70 mg/kg/day. (B) p values from the statistical assessment of the additional contribution of dantrolene to e23AON treatment for exon 23 skipped/(skipped + unskipped) transcript as measured by ddPCR. p values are from Friedman's test, a two-way nonparametric ANOVA, and a meta-analysis of the results across muscles.

e23AON relative to e23AON alone based on dystrophin immunofluorescence ($p = 0.0025$) and dystrophin-positive fibers ($p = 0.00028$, Figure 4C). The effect size is considerable in some instances. For example, at 300 mg/kg e23AON, the dantrolene co-administration resulted in an average doubling of dystrophin fluorescence from 13.9% to 27.5% and percentage of positive fibers from 12.6% to 23.1% of levels observed in control C57Bl6 mice in the quadriceps (Figures 4A and 4B).

Combination therapy boosted dystrophin protein expression relative to e23AON treatment alone, as measured by western blot analysis of total protein in the quadriceps (Figures 5A and 5B), with a high level of significance ($p = 0.0054$, Figures 5C and S5). Increases were on the order of 1%–1.7% of the amount of dystrophin typically observed in non mutant C57Bl6 mice quadriceps, distributed across a subset of fibers (Figure 4D).

To determine whether the increase in dystrophin observed with co-administration of dantrolene helps promote sarcolemmal DGC protein stability, we assessed the effect of dantrolene on other members of the DGC. Long-term dantrolene combined with e23AON administration rescued the sarcolemmal localization of both α-sarcoglycan and β-dystroglycan, indicators of DGC rescue. Figure 6 shows images from the 50 mg/kg e23AON treatment group (Figure 6); similar results were seen at 10 mg/kg and 300 mg/kg e23AON (data not shown). Immunostaining of skeletal muscle demonstrated positive staining at the N terminus, rod domain, and C terminus of dystrophin (Figure S6). Serial sections of the quadriceps and diaphragm indicate that dystrophin-positive fibers colocalized with fibers expressing DGC proteins. Together, these findings demonstrate that dantrolene boosts e23AON activity during long-term treatment to facilitate restoration of dystrophin protein, with sufficient functionality to enable DGC rescue and localization to the sarcolemma.

Dantrolene/e23AON Combination Therapy Reverses Pathology to a Greater Extent than e23AON Alone

Serum creatine kinase (CK) levels are an indicator of muscle fiber integrity because muscle CK leaks into the serum upon membrane damage. After 6 months of treatment, serum CK levels in *mdx* mice were reduced in response to e23AON, as expected. These levels were further reduced significantly with daily oral dantrolene supplementation across all e23AON treatments combined ($p < 0.0001$, Figures 7A, 7B and S7). In keeping with a previous report, *mdx* treatment with 40 mg/kg dantrolene alone lowers CK to some extent but not to the same degree as treatment with dantrolene and e23AON combined.[31] However, the high degree of rescue induced by each dantrolene and phosphorodiamidate morpholino oligomer (PMO) alone precludes distinguishing between a synergistic versus additive effect.

A

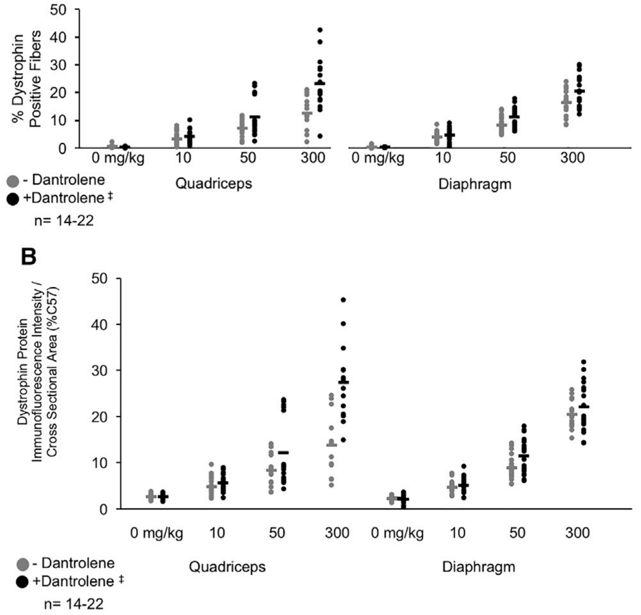

C

Outcome Measure	Relative Fold Change with Dantrolene Treatment	Friedman's Two-way ANOVA by ranks: Quadriceps (p-value)	Friedman's Two-way ANOVA by ranks: Diaphragm (p-value)	Meta analysis
Dystrophin immunofluorescence quantitation (%C57)	1.33	0.0032	0.086	0.0025
Dystrophin positive fibers (%)	1.42	0.0069	0.0035	0.00028

D

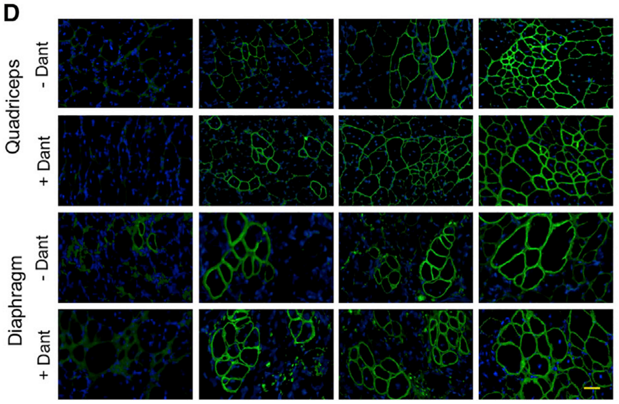

Figure 4. Dantrolene Boosts e23AON to Promote Rescue of Dystrophin Immunofluorescence Intensity and Percentage of Dystrophin-Positive Fibers

(A) Effect of dantrolene on e23AON-induced skipped dystrophin protein as measured by quantitative immunofluorescence of individual quadriceps and diaphragms stained with an anti-dystrophin antibody (MANDYS8) that recognizes the rod domain of dystrophin. One cross-section per muscle per animal was evaluated for total dystrophin signal above a minimum background threshold and normalized to total cross-sectional area as described in the methods. Data are presented as a percentage of C57 control levels of dystrophin. (B) Effect of dantrolene on e23AON-induced expression of dystrophin protein in mdx mice treated for 6 months as measured by % dystrophin-positive fibers in individual quadriceps and diaphragms. One cross-section per muscle per animal was evaluated for dystrophin expression. ‡Dantrolene was dosed at 30–70 mg/kg/day. (C) p values from the multivariate assessment of the effect of dantrolene on e23AON treatment for dystrophin immunofluorescence quantitation and dystrophin-positive fibers. p values from the statistical assessment of the additional contribution of dantrolene to e23AON treatment for exon 23 skipped/(skipped + unskipped) transcript as measured by ddPCR. p values are from Friedman's test, a two-way nonparametric ANOVA, and a meta-analysis of the results across muscles for dystrophin immunofluorescence quantitation. (D) Effect of dantrolene on e23AON sarcolemmal dystrophin expression as illustrated by representative images from the quadriceps and diaphragms of mdx mice after treatment with e23AON ± dantrolene. Scale bar, 50 μm.

To determine the effect of combination therapy on *mdx* muscle histopathology, we quantitated (1) ongoing regeneration of myofibers by assessing embryonic myosin heavy chain (eMHC) expression and (2) degeneration/regeneration by assessing myofiber centronucleation.[32–37] In the *mdx* model, muscle injury leads to increases in regeneration/degeneration, and eMHC and centronucleation are each anticipated to decrease with dystrophin rescue. eMHC and centronucleation were both decreased in response to e23AON treatment alone (Figures 8A, 8D, and S8).

Dantrolene co-administration caused a decrease in centronucleation in the diaphragm to a high degree of certainty when examined across all e23AON treatments combined ($p = 0.017$, Figures 8A, 8D, and S8). Although statistical significance was not observed when centronucleation in the quadriceps was analyzed independently, there was a trend toward less centronucleation when analyzed together ($p = 0.066$). Dantrolene, when co-administered with e23AON, is also observed to cause a decrease in eMHC-positive fibers relative to e23AON alone in the quadriceps ($p = 0.021$) and diaphragm ($p = 0.025$) to a high degree of certainty based on the multivariate analysis (Figures 8B–8D and S8). Meta-analysis of the p values from the quadriceps and diaphragm combined supports an effect from dantrolene/e23AON treatment relative to e23AON alone on both centronucleated fibers ($p = 0.0087$) and eMHC-positive fibers ($p = 0.0045$, Figure 4C). These findings demonstrate that dantrolene boost can impact *mdx* pathophysiology, which is predicted to impact muscle function, and highlights the potential of combination therapy to improve therapeutic outcome.

Meta-Analyses Show that Dantrolene Significantly Boosts the AON Effect on All Quantitative Outcomes

The levels of significance of dantrolene boost across both muscles are shown side by side in Table 1. Taken together, our meta-analyses demonstrate that dantrolene boosts the e23AON effect on all quantitative outcomes of exon skipping, dystrophin expression, and pathophysiology measured when considered across both muscles combined, with a high level of significance ($p = 0.00012–0.0087$).

DISCUSSION

Here, we provide preclinical mouse data in support of re-purposing dantrolene to boost AON-guided *DMD* exon skipping. Dantrolene is already FDA approved for both malignant hyperthermia and spasticity[38] and has been used broadly in the pediatric population. When

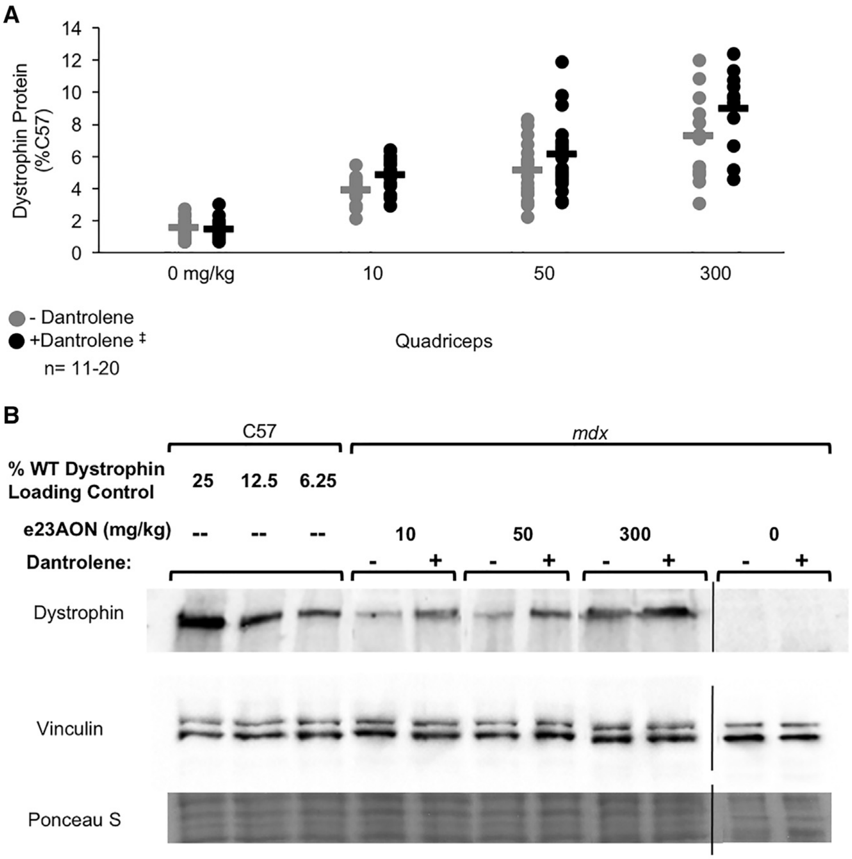

C

		Friedman's Two-way ANOVA by ranks
Outcome Measure	Relative Fold Change with Dantrolene Treatment	Quadriceps (p-value)
Dystrophin protein by Western blot (%C57)	1.23	0.0054

Figure 5. Dantrolene Boosts e23AON to Promote Rescue of Dystrophin Protein after Chronic Treatment

(A) Western blot demonstrating the effect of dantrolene on e23AON-induced expression of total dystrophin protein in mdx mice treated for 6 months. Protein from each muscle of mice in all treatments group was isolated and quantified independently. ‡Dantrolene was dosed at 30–70 mg/kg/day. (B) Representative western blots for dystrophin protein expression from quadriceps are shown. C57BL/6 protein was loaded at 25%, 12.5%, and 6.25% dilutions (diluted in protein samples from mdx mice to serve as a standard). (C) p values from the multivariate assessment of the effect of dantrolene on e23AON treatment for dystrophin protein by western blot. p values are from Friedman's test, a two-way nonparametric ANOVA, and a meta-analysis of the results across muscles.

used in combination with e23AON, 30–70 mg/kg/day dantrolene modestly increased e23-skipped mRNA, dystrophin protein expression, and rescue of pathophysiology after 6 months of chronic dosing in the *mdx* DMD mouse model. We used a dosing scheme more compatible with human DMD treatment than our previous studies,[25] which relied on 2X daily i.p. dantrolene injection, attempting to more closely approximate a regimen of chronic oral dosing of dantrium in humans.

Although no published study exactly recapitulates our dosing scheme, two published studies report a higher degree of dystrophin rescue in mdx mice than reported here after long-term treatment with similar doses of PMO.[26,27] The reason for this is unclear. Possible explanations for differences observed between studies include timing of dosing, length of study, quantitation or injection of the input morpholino, or sensitivity of the methods used to measure dystrophin. Of note, the dystrophin output reported in the two published studies also vary by two-fold, implying that either dosing or differences in laboratory protocol can impact reported rescued dystrophin levels.[26,27] Regardless, because we observed a significant dantrolene boost effect across a wide range of PMO doses, such differences do not greatly impact the conclusion reached that dantrolene has observed efficacy as a skip booster at a range of PMO doses and dystrophin induction. The modest 32%–37% relative fold change with dantrolene treatment reported in Table 1 is averaged across all e23AON concentrations tested, including no AON, which underestimates the true effect size at a given AON dose. There is an apparent correlation that at higher doses of e23AON, there was a large effect of dantrolene co-administration.

Mouse to human equivalence formulas would predict that a 30 mg/kg/week Exondys51 dosing in boys weighing 20 kg would be equivalent to 240 mg/kg weekly dosing in mice.[39] However, the AON exon-skipping drug appears not to scale typically from mouse to dog using standard equivalence formulas based on surface area and therefore is unlikely to scale typically in humans.[40] In our experiments, the 300 mg/kg e23AON dose induces 7.2% dystrophin, distributed across 12.6% of the quadriceps fibers. By comparison, ExonDys51 induces significantly less dystrophin in DMD boys, on average 0.93% (range, 0%–2.47%), distributed across 16% of fibers (range, 1.4%–33.5%[15–21]). At 50 mg/kg e23AON, we observed, on average, 5.1% dystrophin distributed across 7.1% of quadriceps fibers, whereas 10 mg/kg yields 3.9% dystrophin across 3.4% of fibers. Although none of these doses perfectly approximate the reported data from humans treated with 30–50 mg/kg/week, based on measures of dystrophin rescue, we have demonstrated a dantrolene effect across doses that span a broad range of AON, including concentrations most likely to recapitulate approved dosing of 30 mg/kg/week Exondys51. Therefore, dantrolene promotes skipping activity in the mouse model and has high potential in humans when used in combination with

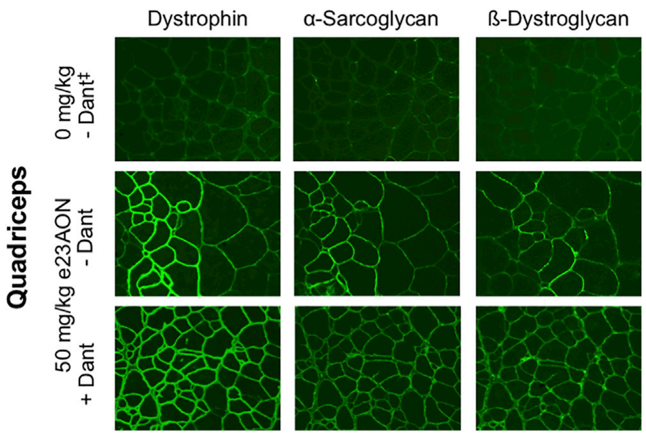

Figure 6. Dantrolene/e23AON Combination Therapy Restores Sarcolemmal Dystrophin and DGC after Chronic Treatment

Effect of dantrolene/e23AON combination therapy on rescue of DGC components as illustrated by representative immunofluorescence images of serial cross-sections from treated mdx quadriceps. Dystrophin was detected with an antibody specific to the rod domain (MANDYS8). Additional DGC components, α-sarcoglycan and β-dystroglycan, were detected with NCL-α-SARC and NCL-β-DG antibodies, respectively. ‡Dantrolene was dosed at 30–70 mg/kg/day.

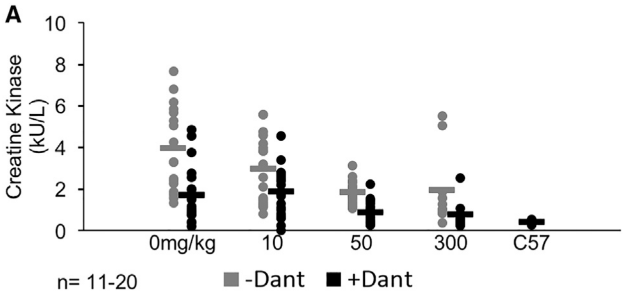

B

		Friedman's Two-way ANOVA by ranks
Outcome Measure	Relative Fold Change with Dantrolene Treatment	Serum (p-value)
Serum CK	0.49	<0.0001

Figure 7. Creatine Kinase Reduction Is Boosted with Dantrolene/e23AON Combination Therapy

(A) A reduction in creatine kinase levels with the addition of dantrolene in mice receiving 0, 10, and 50 mg/kg e23AON/dantrolene combination. (B) p values from the multivariate assessment of the effect of dantrolene on e23AON treatment for serum CK levels. p values from the statistical assessment of the additional contribution of dantrolene to e23AON treatment for exon 23 skipped/(skipped + unskipped) transcript as measured by ddPCR. p values are from Friedman's test, a two-way nonparametric ANOVA, and meta-analysis of the results across muscles.

currently available Exondys51 dosing. However, it will be important to test dantrolene/Exondys51 efficacy in the context of a human DMD trial to establish safety and efficacy prior to wide adoption and implementation in clinical practice.

The pivotal Exondys51 trial used to support accelerated approval relied on 30–50 mg/kg weekly dosing in humans and is consistent with a functional benefit in boys with DMD.[17] At accelerated approval, the FDA suggested that exploring higher and/or more frequent Exondys51 dosing to increase the amount of dystrophin induced and better observe clinical efficacy is necessary. However, increasing the frequency of dosing of Exondys51 is unlikely to be practical, and it is unclear if higher dosing is feasible from a toxicity perspective.[41] AON modifications that promise better muscle cell uptake, such as peptide linkages, and that use skip boosting agents represent alternative approaches to increasing dystrophin levels. In addition to our identifying dantrolene boost activity,[25] others have reported skip boosting using CELF2a inhibitors, 6-thioguanine, NOL8 protein, and carbohydrate-based infusions.[40,42–45] However, dantrolene is the only FDA-approved drug with previous exposure in boys with DMD and a good safety profile.

We have previously published that dantrolene boosts AON exon 51 skipping in human patient fibroblast-derived myotube cultures.[25] Our published findings showing that dantrolene can boost skipping in combination with either AON or 2-o-methyl AON chemistries may indicate that dantrolene boost is agnostic to AON chemistry.[25]

Dantrolene is an FDA-approved drug indicated for chronic use for spasticity (1.6–6.5 mg/kg/day[38]) and acutely for malignant hyperthermia (4–8 mg/kg[38]) and has been widely studied in multiple animal models and humans.[46–50] It targets the RyR1, which is responsible for Ca^{2+} release from the sarcoplasmic reticulum (SR) and regulating levels of cytosolic and SR luminal Ca^{2+} levels essential for excitation-contraction coupling.

In the current study, we measured dantrolene serum levels to be an average of 0.26 μg/mL ± 0.18, which is at the low end of the known therapeutic range used in humans (0.37 to 1.24 μg/mL).[46,51,52] Together with findings that other RyR calcium modulators, like RyCal S107, can also boost exon skipping, it is likely dantrolene boost activity results from RyR calcium modulation.[53–55] Calcium regulation of alternative splicing has been described in other models.[56–58]

In DMD, RyR has been shown to be dysregulated and contributes to increased intracellular calcium and DMD pathology.[5] As a potential means of controlling calcium-handling defects in DMD, Bertorini et al.[59] administered 8 mg/kg/day dantrolene to 7 DMD boys over a 2-year period. In those studies, dantrolene was well tolerated in 6/7 patients; one boy experienced increased, but mild, muscle weakness that was reversed upon lowering the dose to 6 mg/kg/day. A trend toward reducing manual muscle testing deterioration was observed, and a statistically significant reduction in CK levels was reported. Although the data were insufficient to establish a clear functional benefit, this study demonstrates long-term treatment with dantrolene can be tolerated in children with DMD.

We demonstrated reduced CK levels in mdx mice treated with dantrolene alone, but observed no effect on any other measures of mdx histopathology or exon skipping in the absence of e23AON,

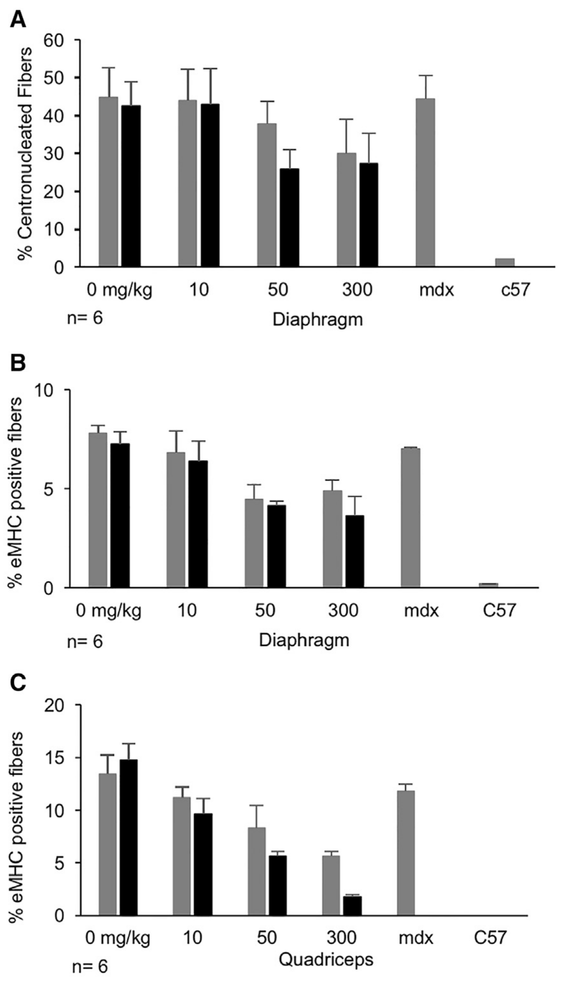

D

Outcome Measure	Relative Fold Change with Dantrolene Treatment	Friedman's Two-way ANOVA by ranks		Meta analysis
		Quadriceps (p-value)	Diaphragm (p-value)	
Centronucleated fibers (%)	0.97	0.066	0.017	0.0087
eMHC positive fibers (%)	0.83	0.021	0.025	0.0045

Figure 8. Dantrolene/AON Combination Therapy Reduces Centronucleation in the Diaphragm and eMHC-Positive Fibers in the Quadriceps

(A) Cross-sections of diaphragm muscles were stained with H&E, highlighting centronucleated fibers, and the percentage of fibers with centrally located nuclei was counted. Cross-sections of diaphragm (B) and quadriceps (C) muscles were stained with anti-eMHC, highlighting regenerating fibers. ‡Dantrolene was dosed at 30–70 mg/kg/day. (D) p values from the multivariate assessment of the effect of dantrolene on e23AON treatment for centronucleation and eMHC-positive fibers. p values from the statistical assessment of the additional contribution of dantrolene to e23AON treatment for exon 23 skipped/(skipped + unskipped) transcript as measured by ddPCR. p values are from Friedman's test, a two-way nonparametric ANOVA, and meta-analysis of the results across muscles. Error bars indicate one SD.

consistent with published data.[31,60] In one published study in mdx mice, 6-week 40 mg/kg dantrolene treatment reduced both CK levels and some, but not all, measures of strength, despite no observed effect on histopathology.[31] It is reasonable to anticipate that if there is a beneficial effect in humans, chronic administration will be necessary. Because we observed skip boosting activity at doses in the low range used chronically for spasticity and higher dosing has been reported to induce mild muscle weakness in some instances,[61] it would be prudent to perform a dose-ranging trial in humans prior to routine use of combination therapy for DMD.

In our studies, dantrolene boosts dystrophin levels by as much as 1%–1.7% of normal levels and allows expression in an additional 4%–10% of the fibers at the most relevant AON doses. Our findings provide pre-clinical support and guidance for combination AON/dantrolene therapy for enhancement of Exondys51 or other skipping drugs in the research pipeline for DMD and perhaps other diseases to be targeted by exon skipping. Because Exondys51 and dantrolene are already FDA approved, our findings can be assessed rapidly in human trials, highlighting the value of screening FDA-approved drugs for combination therapies that promise greater efficacy.

MATERIALS AND METHODS

Long-Term Treatment Blinding

The present randomized, blinded, placebo-controlled study was performed on 180 *mdx* mice. Factorial randomization was used and mice were stratified into treatment groups based on animal age and weight and randomly assigned to each treatment group. 24 or 18 *mdx* mice aged 4–6 weeks were used in each group. Procedures involving all *mdx* mice were approved by the Institutional Animal Care and Use Committee (IACUC) of the University of California, Los Angeles (UCLA). Individuals dosing the mice and performing data-generating assays were blinded for all assays. Once all the data were collected, the blind was lifted for the individual performing statistics.

Mice and Tissue Preparation

Procedures involving all *mdx* mice were approved by the IACUC of UCLA. For 3-week and 6-month experiments with systemic injections of e23AON, the quadriceps were harvested and the right muscle was frozen in optimal cutting temperature compound (OCT) for sectioning and analysis by immunohistochemistry (IHC), whereas the left muscle was cut in half and snap frozen for analysis by western blot and ddPCR. For the diaphragm, one-half of the muscle was designated for IHC and one-fourth was designated for western blot and RT-PCR. Mice and chow were weighed weekly.

AON and Dantrolene Administration to *mdx* Mice

e23AON morpholino[62] (5′-GGCCAAACCTCGGCTTACCTGAA AT-3′; provided by Sarepta Therapeutics) was injected intravenously (retro-orbitally) in 100 μL saline. For 3-week experiments, Revonto (dantrolene sodium NDC 27505-001-65; DSM Pharmaceuticals) was resuspended in water and administered by oral gavage in a 100-μL bolus twice daily. For 6-month experiments, control and experimental feed

Table 1. Friedman's Test, Two-Way Nonparametric ANOVA, of the Addition of Dantrolene to e23AON Treatment for Study Outcomes and Meta-Analyses of the p Values across Muscle Types

Outcome Measure	n	Relative Fold Change with Dantrolene Treatment	Quadriceps (p Value)	Diaphragm (p Value)	Serum (p Value)	Meta-Analysis (p Value)
Exon 23 skipped/(skipped + unskipped) transcript (%)	124	1.37	0.012	0.0008	–	0.00012
Dystrophin immunofluorescence quantitation (% C57)	152	1.33	0.0032	0.086	–	0.0025
Dystrophin-positive fibers (%)	154	1.42	0.0069	0.0035	–	0.00028
Dystrophin protein by western blot (%C57)	131	1.23	0.0054	–	–	–
Centronucleated fibers (%)	47	0.97	0.066	0.017	–	0.0087
eMHC-positive fibers (%)	47	0.83	0.021	0.025	–	0.0045
Serum CK	132	0.49	–	–	<0.0001	–

Shown is a summary of statistical analysis described in Figures 3B, 4C, 5C, 7B, and 8D.

was formulated into 58YP chow at 0, 1,121, and 1,328 ppm. Dantrium from capsules (NDC 0115-4433-1 GlobalRPH) (Lot 10007332) was incorporated into chow and irradiated (Newco Distributors) and mice were given *ad libitum* access to chow. To ensure continued exposure to target levels of dantrolene, mice were weighed at week 13 and chow dantrolene concentration was adjusted to 1,328 ppm, where appropriate. The amount of chow consumed was weighed weekly and dantrolene dose was adjusted by bodyweight.

RNA Isolation, qPCR, and ddPCR

Total RNA was isolated from frozen skeletal muscle with the QIAGEN RNeasy Fibrous Tissue Kit and reverse transcribed to cDNA using the iScript cDNA Synthesis Kit (Bio-Rad). The final concentration of digested total DNA was adjusted to fall within the linear range for the Poisson calculation for the expected number of droplets in the digital PCR. Each sample was partitioned into 15,000 droplets on a DG8 cartridge (Bio-Rad) and each droplet was amplified by PCR using the following protocol: initial denaturation step at 95°C for 10 min, 40 cycles of 94°C for 30 s, and 60°C for 60 s, followed by 98°C for 10 min. Primer-probe sets to assess specific full-length or skipped *Dmd* exon 23 mRNAs have been previously described.[25] For amplification of exons from 22 to 24; the percentage of exon skipping was expressed as the total exon 23 skipped transcript as a percentage of total (skipped + unskipped) *Dmd* transcript. Samples were loaded onto the QX200 droplet reader, and ddPCR data were analyzed with QuantaSoft analysis software.[63] The target concentration in each sample was expressed as copies per ng.

Measurement of Serum Creatine Kinase, γ-Glutamyl Transpeptidase, Creatinine, Total Bilirubin, and Blood Urea Nitrogen

Blood was collected by retro-orbital bleeding of the mice. After clotting, samples were spun at 6,000 rpm at 4°C. Serum was collected, snap frozen in liquid nitrogen, and stored at −80°C. Creatine kinase analysis was performed with the Genzyme Creatine Kinase kit (BioPacific Diagnostic). γ-glutamyl transpeptidase, creatinine, total bilirubin, and blood urea nitrogen measurements were performed by the UCLA Division of Laboratory Animal Medicine (DLAM) Pathology and Laboratory Medicine Services.

Measurement of Dantrolene Serum Levels

Blood was collected by retro-orbital bleeding of the mice. After clotting, samples were spun at 6,000 rpm at 4°C. Serum was collected, snap frozen in liquid nitrogen, and stored at −80°C. Dantrolene serum analysis was performed with the Dantrolene ELISA Kit (Neogen; Product #106319; Lot 140923). Samples were run in duplicate. A standard curve was run on each plate to ensure sensitivity of the assay (Figure S2B). Diluted dantrolene solution was used to generate a standard curve.

Western Blot Protocol

Total protein was isolated from flash-frozen quadriceps and diaphragms from the treated *MDX* and control C57 mice. Tissues were homogenized in Mito buffer (0.2 mM EDTA, 0.25 mM sucrose, and 10 mM tris-HCl [pH 7.4]) with protease/phosphatase inhibitor cocktail (Pierce) and deoxyribonuclease/ribonuclease and subjected to low-speed (3,000 × *g*) centrifugation for 10 min at 4°C. The supernatant was centrifuged at 100,000 × *g* (high-speed centrifugation) for 1 hr for isolation of membrane fraction. Isolated membranes and pellet after low-speed centrifugation were combined and resuspended in 300 mL extraction buffer (50 mM tris-HCl [pH 7.4], 7 M urea, 2 M thiourea, 4% 3-[(3-cholamidopropyl)dimethylammonio]-1-propanesulfonate (CHAPS), 2% SDS, and 50 mM b-mercaptoethanol). Protein concentration was determined by 2-D Quant Kit (GE Healthcare Life Sciences).

40 μg total protein were run on a 6% polyacrylamide gel and transferred onto a nitrocellulose membrane for 2 hr at 4°C. The membrane was blocked for 1 hr in 5% milk and then incubated with MANDYS8 (Sigma) (anti-dystrophin), 1:400 in Tris-buffered saline with tween (TBS-T), hVin-1 (Sigma), 1:5,000 in TBST (antivinculin), and a skeletal muscle membrane protein not associated with the DGC that was used as a loading control.

For analysis, dystrophin protein levels were normalized to the loading and then pooled across treatment groups to determine the average dystrophin rescue. Serial dilutions of C57 sample into untreated *MDX* sample were run simultaneously. All western data included were run through quality control, as defined by linearity of dilution of C57 controls. The average densitometry value for 100% of dystrophin in C57 was calculated as the mean of the densitometry values of C57 serial dilutions multiplied by the dilution factor. Densitometry analysis was performed with the ImageLab 5.1 gel documentation system (Bio-Rad).

Histology and Immunofluorescence

IHC was performed on unfixed 10-mm tissue sections using the MouseOnMouse kit (Vector Labs). IHC assessment used the following primary antibodies: MANDYS8 (dystrophin rod domain), Ab15277 (dystrophin C terminus; Abcam), and Manex1A (dystrophin N terminus; Developmental Studies Hybridoma Bank). α-Sarcoglycan was detected with NCL-α-SARC (Novocastra), and β-dystroglycan was detected with NCL-β-DG (Novocastra), eMHC (DSHB; BF-G6-c), and DNA with DAPI. Secondary labeling was performed with fluorescein isothiocyanate (FITC) anti-mouse or FITC anti-rabbit (Vector Labs). Sections were mounted in Vectashield Mounting Medium (Vector Labs). Fluorescent images were acquired and analyzed using Ariol SL-50 (Applied Imaging, San Jose, CA). The Ariol scanner is based on an Olympus BX61 microscope with motorized stage and autofocus capabilities, equipped with a black and white video camera (Jai CVM2CL). Scanning and analyses were performed through the Translational Pathology Core Laboratory, Department of Pathology and Laboratory Medicine, David Geffen School of Medicine at UCLA. For quantifying dystrophin immunofluorescence, the minimum threshold was placed above background level for all sections. Analytical readouts included area of immunofluorescence (IFL) signal (area of signal above the signal threshold) and total area of the section. The total area of positive pixels above the minimum threshold per muscle section was counted using AxioVision Rel.4.6.3.0 acquisition software. Each sample's dystrophin immunofluorescent levels are expressed as a percentage of area of positive pixels in C57 (100%). For quantifying dystrophin-positive fibers, the minimum threshold was placed above background level for all sections. For each sample, fibers were counted across the entire section. Dystrophin-positive fibers were defined as fibers that had sarcolemmal dystrophin expression above background and surrounding >75%–80% of each fiber. For both measurements, all of our readings were well below the maximal intensity threshold (pixel saturation was avoided). For detecting DGC-associated proteins by IHC, 2 mice from each treatment group, including non-treated *mdx* controls, were selected and the samples were blinded. Samples were stained and entire cross-sections were visualized. While blinded, representative fields for each sample were taken. A subset of these images is included as Figure 6. For quantifying centronucleation, eMHC-positive fibers, diaphragms, and quadriceps from the 3 to 6 mice per treatment group, which had total dystrophin levels by western blot near the median, were selected. The average percentage of fibers with centrally located nuclei in the diaphragm per group was determined by counting entire muscle cross-sections. For quantifying eMHC-positive fibers, quadriceps/diaphragms from 6 mice per group, which had total dystrophin levels by western blot near the median, were selected. The total number of eMHC-positive fibers were counted and compared to the total number of muscle fibers per section. For pathology, a standard protocol was used for H&E staining. All mouse livers, hearts, and kidneys were reviewed blindly by a certified pathologist.

Statistical Analysis

We selected to test whether dantrolene makes an additional contribution to the outcomes beyond E23AON using a 2-way ANOVA with interactions. However, we observed that the outcomes data are not normally distributed, and performed a nonparametric version of analysis using Friedman's test using SAS software (version 9.4, SAS Institute, Cary, NC). We ran each analysis on the ranks of the observations for each of the 7 traits (or assays). Quadriceps, diaphragm, and serum were analyzed separately. Each test included E23AON first, followed by dantrolene and the possibility of their interaction. None of the interactions were significant. The separate outcome results were combined by meta-analyses across muscle types, resulting in tests of 6 non-independent traits. A 0.01 level of significance was set to account for multiple testing of 6 traits that are not independent. For each outcome, E23AON was highly significantly ($p < 0.0001$), except for centronucleated fibers in the quadriceps ($p < 0.04$) (data not shown). Table 1 presents the p values for dantrolene for each of the outcomes. A standard protocol was used for H&E staining. The average percentage of fibers with centrally located nuclei in the diaphragm per group was determined by counting 750–1,000 fibers from the 3 mice per group, which had total dystrophin levels by western blot nearest the median. All mouse livers, hearts, and kidneys were reviewed blindly.

SUPPLEMENTAL INFORMATION

Supplemental Information includes eight figures and can be found with this article online at https://doi.org/10.1016/j.omtn.2018.02.002.

AUTHOR CONTRIBUTIONS

D.W.W. led the implementation of mouse experiments and performed the RNA skipping quantification experiments, pathophysiology experiments, and serum biomarker experiments. E.I.M. performed muscle sectioning and quantitative immunohistochemistry and western blotting for dystrophin. Y.B.N. performed pathological assessment. D.W.W. contributed as first author. E.I.M., G.C.K., and D.B. provided technical support. D.W.W., M.C.M., and S.F.N. wrote the manuscript with input from the other authors. M.C.M. and S.F.N. conceived the project, designed the experiments, and supervised the entire study. M.J.S. helped implement the mouse dosing together with M.C.M. and S.F.N. R.M.C. conceived the statistical model and meta-analyses used in the study. M.C.M. and S.F.N. contributed equally as senior authors.

CONFLICTS OF INTERESTS

S.F.N., M.C.M., and M.M. are inventors on a pending patent on identification of small molecules that enhance exon skipping filed by UCLA.

ACKNOWLEDGMENTS

We thank J. Wen and L. Martinez for providing technical support. We thank F. Barthélémy for critical reading and providing expert input throughout the project. Scanning, quantitative analysis of muscle cross-sections, and staining and histological analysis of organs were performed at the UCLA Translational Pathology Core Laboratory. This work was primarily funded from grants from the NIH (1T32AR65972A1) and the California Institute of Regenerative Medicine (TRX-05426). Support of the research was also provided by the UCLA Muscular Dystrophy Core Center (NIH 5P30AR057230) within the Center for Duchenne Muscular Dystrophy at UCLA.

REFERENCES

1. Mah, J.K., Korngut, L., Dykeman, J., Day, L., Pringsheim, T., and Jette, N. (2014). A systematic review and meta-analysis on the epidemiology of Duchenne and Becker muscular dystrophy. Neuromuscul. Disord. *24*, 482–491.
2. Emery, A.E. (2002). The muscular dystrophies. Lancet *359*, 687–695.
3. Biressi, S., Miyabara, E.H., Gopinath, S.D., Carlig, P.M., and Rando, T.A. (2014). A Wnt-TGFβ2 axis induces a fibrogenic program in muscle stem cells from dystrophic mice. Sci. Transl. Med. *6*, 267ra176.
4. Tanihata, J., and Takeda, S. (2016). [Changes in cytosolic Ca2+dynamics associated with muscular dystrophy]. Clin. Calcium *26*, 1677–1683.
5. Bellinger, A.M., Reiken, S., Carlson, C., Mongillo, M., Liu, X., Rothman, L., Matecki, S., Lacampagne, A., and Marks, A.R. (2009). Hypernitrosylated ryanodine receptor calcium release channels are leaky in dystrophic muscle. Nat. Med. *15*, 325–330.
6. Samaha, F.J., and Quinlan, J.G. (1996). Dystrophinopathies: clarification and complication. J. Child Neurol. *11*, 13–20.
7. Emery, A.E., and Skinner, R. (1976). Clinical studies in benign (Becker type) X-linked muscular dystrophy. Clin. Genet. *10*, 189–201.
8. Nicolas, A., Lucchetti-Miganeh, C., Yaou, R.B., Kaplan, J.C., Chelly, J., Leturcq, F., Barloy-Hubler, F., and Le Rumeur, E. (2012). Assessment of the structural and functional impact of in-frame mutations of the DMD gene, using the tools included in the eDystrophin online database. Orphanet J. Rare Dis. *7*, 45.
9. Mann, C.J., Honeyman, K., Cheng, A.J., Ly, T., Lloyd, F., Fletcher, S., Morgan, J.E., Partridge, T.A., and Wilton, S.D. (2001). Antisense-induced exon skipping and synthesis of dystrophin in the mdx mouse. Proc. Natl. Acad. Sci. USA *98*, 42–47.
10. Foster, H., Popplewell, L., and Dickson, G. (2012). Genetic therapeutic approaches for Duchenne muscular dystrophy. Hum. Gene Ther. *23*, 676–687.
11. Kole, R., and Krieg, A.M. (2015). Exon skipping therapy for Duchenne muscular dystrophy. Adv. Drug Deliv. Rev. *87*, 104–107.
12. Yokota, T., Lu, Q.L., Partridge, T., Kobayashi, M., Nakamura, A., Takeda, S., and Hoffman, E. (2009). Efficacy of systemic morpholino exon-skipping in Duchenne dystrophy dogs. Ann. Neurol. *65*, 667–676.
13. Alter, J., Lou, F., Rabinowitz, A., Yin, H., Rosenfeld, J., Wilton, S.D., Partridge, T.A., and Lu, Q.L. (2006). Systemic delivery of morpholino oligonucleotide restores dystrophin expression bodywide and improves dystrophic pathology. Nat. Med. *12*, 175–177.
14. Lu, Q.L., Mann, C.J., Lou, F., Bou-Gharios, G., Morris, G.E., Xue, S.A., Fletcher, S., Partridge, T.A., and Wilton, S.D. (2003). Functional amounts of dystrophin produced by skipping the mutated exon in the mdx dystrophic mouse. Nat. Med. *9*, 1009–1014.
15. Kinali, M., Arechavala-Gomeza, V., Feng, L., Cirak, S., Hunt, D., Adkin, C., Guglieri, M., Ashton, E., Abbs, S., Nihoyannopoulos, P., et al. (2009). Local restoration of dystrophin expression with the morpholino oligomer AVI-4658 in Duchenne muscular dystrophy: a single-blind, placebo-controlled, dose-escalation, proof-of-concept study. Lancet Neurol. *8*, 918–928.
16. Mendell, J.R., Rodino-Klapac, L.R., Sahenk, Z., Roush, K., Bird, L., Lowes, L.P., Alfano, L., Gomez, A.M., Lewis, S., Kota, J., et al.; Eteplirsen Study Group (2013). Eteplirsen for the treatment of Duchenne muscular dystrophy. Ann. Neurol. *74*, 637–647.
17. Mendell, J.R., Goemans, N., Lowes, L.P., Alfano, L.N., Berry, K., Shao, J., Kaye, E.M., and Mercuri, E.; Eteplirsen Study Group and Telethon Foundation DMD Italian Network (2016). Longitudinal effect of eteplirsen versus historical control on ambulation in Duchenne muscular dystrophy. Ann. Neurol. *79*, 257–271.
18. Peripheral and Central Nervous System Drugs Advisory Committee. (2016). Eteplirsen briefing document. NDA 206488, April 25, 2016. https://www.fda.gov/downloads/advisorycommittees/committeesmeetingmaterials/drugs/peripheralandcentralnervoussystemdrugsadvisorycommittee/ucm497064.pdf
19. Peripheral and Central Nervous System Drugs Advisory Committee Meeting. (2016). FDA briefing document. NDA 206488 eteplirsen, April 25, 2016. https://www.fda.gov/downloads/advisorycommittees/committeesmeetingmaterials/drugs/peripheralandcentralnervoussystemdrugsadvisorycommittee/ucm497063.pdf.
20. Unger, E.F., and Califf, R.M. (2017). Regarding "Eteplirsen for the treatment of Duchenne muscular dystrophy". Ann. Neurol. *81*, 162–164.
21. Mendell, J.R. (2017). Reply. Ann. Neurol. *81*, 164–165.
22. Hoffman, E.P., Fischbeck, K.H., Brown, R.H., Johnson, M., Medori, R., Loike, J.D., Harris, J.B., Waterston, R., Brooke, M., Specht, L., et al. (1988). Characterization of dystrophin in muscle-biopsy specimens from patients with Duchenne's or Becker's muscular dystrophy. N. Engl. J. Med. *318*, 1363–1368.
23. van den Bergen, J.C., Wokke, B.H., Janson, A.A., van Duinen, S.G., Hulsker, M.A., Ginjaar, H.B., van Deutekom, J.C., Aartsma-Rus, A., Kan, H.E., and Verschuuren, J.J. (2014). Dystrophin levels and clinical severity in Becker muscular dystrophy patients. J. Neurol. Neurosurg. Psychiatry *85*, 747–753.
24. van Putten, M., Hulsker, M., Young, C., Nadarajah, V.D., Heemskerk, H., van der Weerd, L., 't Hoen, P.A., van Ommen, G.J., and Aartsma-Rus, A.M. (2013). Low dystrophin levels increase survival and improve muscle pathology and function in dystrophin/utrophin double-knockout mice. FASEB J. *27*, 2484–2495.
25. Kendall, G.C., Mokhonova, E.I., Moran, M., Sejbuk, N.E., Wang, D.W., Silva, O., Wang, R.T., Martinez, L., Lu, Q.L., Damoiseaux, R., et al. (2012). Dantrolene enhances antisense-mediated exon skipping in human and mouse models of Duchenne muscular dystrophy. Sci. Transl. Med. *4*, 164ra0.
26. Malerba, A., Sharp, P.S., Graham, I.R., Arechavala-Gomeza, V., Foster, K., Muntoni, F., Wells, D.J., and Dickson, G. (2011). Chronic systemic therapy with low-dose morpholino oligomers ameliorates the pathology and normalizes locomotor behavior in mdx mice. Mol. Ther. *19*, 345–354.
27. Wu, B., Xiao, B., Cloer, C., Shaban, M., Sali, A., Lu, P., Li, J., Nagaraju, K., Xiao, X., and Lu, Q.L. (2011). One-year treatment of morpholino antisense oligomer improves skeletal and cardiac muscle functions in dystrophic mdx mice. Mol. Ther. *19*, 576–583.
28. Kilkenny, C., Parsons, N., Kadyszewski, E., Festing, M.F., Cuthill, I.C., Fry, D., Hutton, J., and Altman, D.G. (2009). Survey of the quality of experimental design, statistical analysis and reporting of research using animals. PLoS One *4*, e7824.
29. Friedman, M. (1939). A correction: The use of ranks to avoid the assumption of normality implicit in the analysis of variance. J. Am. Stat. Assoc. *34*, 109.
30. Friedman, M. (1937). The use of ranks to avoid the assumption of normality implicit in the analysis of variance. J. Am. Stat. Assoc. *32*, 675–701.
31. Quinn, J.L., Huynh, T., Uaesoontrachoon, K., Tatem, K., Phadke, A., Van der Meulen, J.H., Yu, Q., and Nagaraju, K. (2013). Effects of dantrolene therapy on disease phenotype in dystrophin deficient mdx mice. PLoS Curr. 5.
32. Enwere, E.K., Boudreault, L., Holbrook, J., Timusk, K., Earl, N., LaCasse, E., Renaud, J.M., and Korneluk, R.G. (2013). Loss of cIAP1 attenuates soleus muscle pathology and improves diaphragm function in mdx mice. Hum. Mol. Genet. *22*, 867–878.
33. Karpati, G., Carpenter, S., and Prescott, S. (1988). Small-caliber skeletal muscle fibers do not suffer necrosis in mdx mouse dystrophy. Muscle Nerve *11*, 795–803.
34. Malerba, A., Thorogood, F.C., Dickson, G., and Graham, I.R. (2009). Dosing regimen has a significant impact on the efficiency of morpholino oligomer-induced exon skipping in mdx mice. Hum. Gene Ther. *20*, 955–965.
35. Janssen, P.M., Murray, J.D., Schill, K.E., Rastogi, N., Schultz, E.J., Tran, T., Raman, S.V., and Rafael-Fortney, J.A. (2014). Prednisolone attenuates improvement of cardiac and skeletal contractile function and histopathology by lisinopril and spironolactone in the mdx mouse model of Duchenne muscular dystrophy. PLoS One *9*, e88360.

36. Kobayashi, Y.M., Rader, E.P., Crawford, R.W., and Campbell, K.P. (2012). Endpoint measures in the mdx mouse relevant for muscular dystrophy pre-clinical studies. Neuromuscul. Disord. *22*, 34–42.

37. Banks, G.B., Combs, A.C., Odom, G.L., Bloch, R.J., and Chamberlain, J.S. (2014). Muscle structure influences utrophin expression in mdx mice. PLoS Genet. *10*, e1004431.

38. Dantrolene sodium [package insert]. 2011, JHP Pharmaceuticals, LLC: Rochester, MI.

39. Food and Drug Administration Center for Drug Evaluation and Research (CDER) (2005). Estimating the maximum safe starting dose in initial clinical trials for therapeutics in adult healthy volunteers. Report of the U.S. Department of Health and Human Services, July 2005.

40. Moulton, H.M., and Moulton, J.D. (2010). Morpholinos and their peptide conjugates: therapeutic promise and challenge for Duchenne muscular dystrophy. Biochim. Biophys. Acta *1798*, 2296–2303.

41. ClinicalTrials.gov. (2016). Safety and dose finding study of NS-065/NCNP-01 in boys with Duchenne muscular dystrophy (DMD). https://clinicaltrials.gov/ct2/show/NCT02740972?term=exon+skipping&cond=DMD&draw=2&rank=18.

42. Han, G., Gu, B., Cao, L., Gao, X., Wang, Q., Seow, Y., Zhang, N., Wood, M.J., and Yin, H. (2016). Hexose enhances oligonucleotide delivery and exon skipping in dystrophin-deficient mdx mice. Nat. Commun. *7*, 10981.

43. Martone, J., Briganti, F., Legnini, I., Morlando, M., Picillo, E., Sthandier, O., Politano, L., and Bozzoni, I. (2016). The lack of the Celf2a splicing factor converts a Duchenne genotype into a Becker phenotype. Nat. Commun. *7*, 10488.

44. Hu, Y., Wu, B., Zillmer, A., Lu, P., Benrashid, E., Wang, M., Doran, T., Shaban, M., Wu, X., and Lu, Q.L. (2010). Guanine analogues enhance antisense oligonucleotide-induced exon skipping in dystrophin gene in vitro and in vivo. Mol. Ther. *18*, 812–818.

45. O'Leary, D.A., Sharif, O., Anderson, P., Tu, B., Welch, G., Zhou, Y., Caldwell, J.S., Engels, I.H., and Brinker, A. (2009). Identification of small molecule and genetic modulators of AON-induced dystrophin exon skipping by high-throughput screening. PLoS One *4*, e8348.

46. Meyler, W.J., Mols-Thürkow, H.W., and Wesseling, H. (1979). Relationship between plasma concentration and effect of dantrolene sodium in man. Eur. J. Clin. Pharmacol. *16*, 203–209.

47. Wuis, E.W., Vree, T.B., and Van Der Kleijn, E. (1990). Pharmacokinetics of intravenously administered dantrolene and its 5-hydroxy metabolite in dogs. Int. J. Clin. Pharmacol. Res. *10*, 203–210.

48. Flewellen, E.H., and Nelson, T.E. (1980). Dantrolene dose response in malignant hyperthermia-susceptible (MHS) swine: method to obtain prophylaxis and therapeusis. Anesthesiology *52*, 303–308.

49. Flewellen, E.H., Nelson, T.E., and Bee, D.E. (1980). Effect of dantrolene on neouromuscular block by d-tubocurarine and subsequent antagonism by neostigmine in the rabbit. Anesthesiology *52*, 126–130.

50. Meyler, W.J., Mols-Thurkow, I., Scaf, A.H., Sargo, S., and Wesseling, H. (1979). The effect of dantrolene sodium on rat skeletal muscle in relation to the plasma concentration. Eur. J. Pharmacol. *53*, 335–342.

51. Flewellen, E.H., Nelson, T.E., Jones, W.P., Arens, J.F., and Wagner, D.L. (1983). Dantrolene dose response in awake man: implications for management of malignant hyperthermia. Anesthesiology *59*, 275–280.

52. Meyler, W.J., Bakker, H., Kok, J.J., Agoston, S., and Wesseling, H. (1981). The effect of dantrolene sodium in relation to blood levels in spastic patients after prolonged administration. J. Neurol. Neurosurg. Psychiatry *44*, 334–339.

53. Kobayashi, S., Yano, M., Suetomi, T., Ono, M., Tateishi, H., Mochizuki, M., Xu, X., Uchinoumi, H., Okuda, S., Yamamoto, T., et al. (2009). Dantrolene, a therapeutic agent for malignant hyperthermia, markedly improves the function of failing cardiomyocytes by stabilizing interdomain interactions within the ryanodine receptor. J. Am. Coll. Cardiol. *53*, 1993–2005.

54. Kobayashi, S., Bannister, M.L., Gangopadhyay, J.P., Hamada, T., Parness, J., and Ikemoto, N. (2005). Dantrolene stabilizes domain interactions within the ryanodine receptor. J. Biol. Chem. *280*, 6580–6587.

55. Oo, Y.W., Gomez-Hurtado, N., Walweel, K., van Helden, D.F., Imtiaz, M.S., Knollmann, B.C., and Laver, D.R. (2015). Essential role of calmodulin in RyR inhibition by dantrolene. Mol. Pharmacol. *88*, 57–63.

56. Mannino, J.L., Kim, W., Wernick, M., Nguyen, S.V., Braquet, R., Adamson, A.W., Den, Z., Batzer, M.A., Collins, C.C., and Brown, K.D. (2001). Evidence for alternate splicing within the mRNA transcript encoding the DNA damage response kinase ATR. Gene *272*, 35–43.

57. Xie, J., and Black, D.L. (2001). A CaMK IV responsive RNA element mediates depolarization-induced alternative splicing of ion channels. Nature *410*, 936–939.

58. An, P., and Grabowski, P.J. (2007). Exon silencing by UAGG motifs in response to neuronal excitation. PLoS Biol. *5*, e36.

59. Bertorini, T.E., Palmieri, G.M., Griffin, J., Igarashi, M., Hinton, A., and Karas, J.G. (1991). Effect of dantrolene in Duchenne muscular dystrophy. Muscle Nerve *14*, 503–507.

60. Quinlan, J.G., Johnson, S.R., and Samaha, F.J. (1990). Dantrolene normalizes serum creatine kinase in MDX mice. Muscle Nerve *13*, 268–269.

61. Allen, G.C., Cattran, C.B., Peterson, R.G., and Lalande, M. (1988). Plasma levels of dantrolene following oral administration in malignant hyperthermia-susceptible patients. Anesthesiology *69*, 900–904.

62. Gebski, B.L., Mann, C.J., Fletcher, S., and Wilton, S.D. (2003). Morpholino antisense oligonucleotide induced dystrophin exon 23 skipping in mdx mouse muscle. Hum. Mol. Genet. *12*, 1801–1811.

63. Hindson, C.M., Chevillet, J.R., Briggs, H.A., Gallichotte, E.N., Ruf, I.K., Hindson, B.J., Vessella, R.L., and Tewari, M. (2013). Absolute quantification by droplet digital PCR versus analog real-time PCR. Nat. Methods *10*, 1003–1005.

OMTN, Volume *11*

Supplemental Information

Repurposing Dantrolene for Long-Term Combination Therapy to Potentiate Antisense-Mediated *DMD* Exon Skipping in the mdx Mouse

Derek W. Wang, Ekaterina I. Mokhonova, Genevieve C. Kendall, Diana Becerra, Yalda B. Naeini, Rita M. Cantor, Melissa J. Spencer, Stanley F. Nelson, and M. Carrie Miceli

Supplementary Material

Repurposing Dantrolene for Long-term Combination Therapy to Potentiate Antisense-Mediated *DMD* Exon-Skipping in the mdx mouse

Supplementary Figure 1. Oral Dantrolene synergizes with systemically delivered e23AON to promote exon 23 skipping and restoration dystrophin expression after 3 weeks treatment.

(a) Effect of Dantrolene on e23AON-induced skipped dystrophin protein as measured by quantitative immunofluorescence of individual quadriceps and diaphragms. Mice were dosed systemically with once weekly e23AON 50 mg/kg. One cross-section per muscle per animal was evaluated for dystrophin expression, and data are presented a percent of C57 control levels of dystrophin. Error bars represent one standard deviation from the mean. n = 4 – 5. ‡ Dantrolene was dosed at 30-70mg/kg/day. (*P≤0.05 compared to e23kAON only control by two-tailed t-test.)

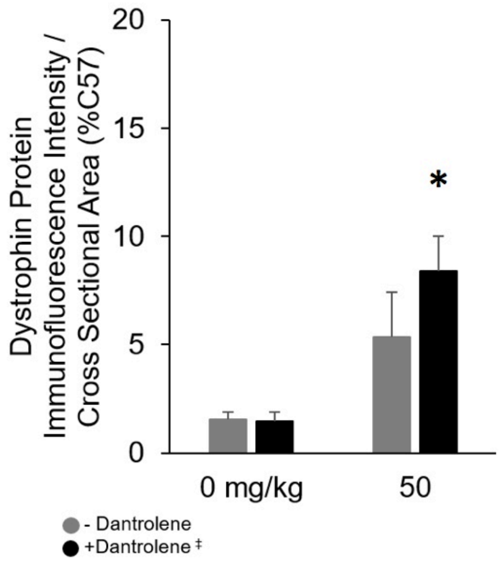

Supplementary Figure 2. Dantrolene was detected at physiologically relevant levels in the serum of mice fed with dantrolene chow.

(a) Serum was isolated from all mice after 26 weeks of treatment. Dantrolene was detected by ELISA. Each sample was run in duplicate. Variability reflects differences between individual mice, not duplicates.To account for 1:5 dilution of sample necessary for dantrolene ELISA, values were multiplied by 5 to calculate final serum levels. n = 60. (b) A standard curve was included on each plate to ensure linearity and all experimentally determined points were within the limits of the dantrolene standard curve.

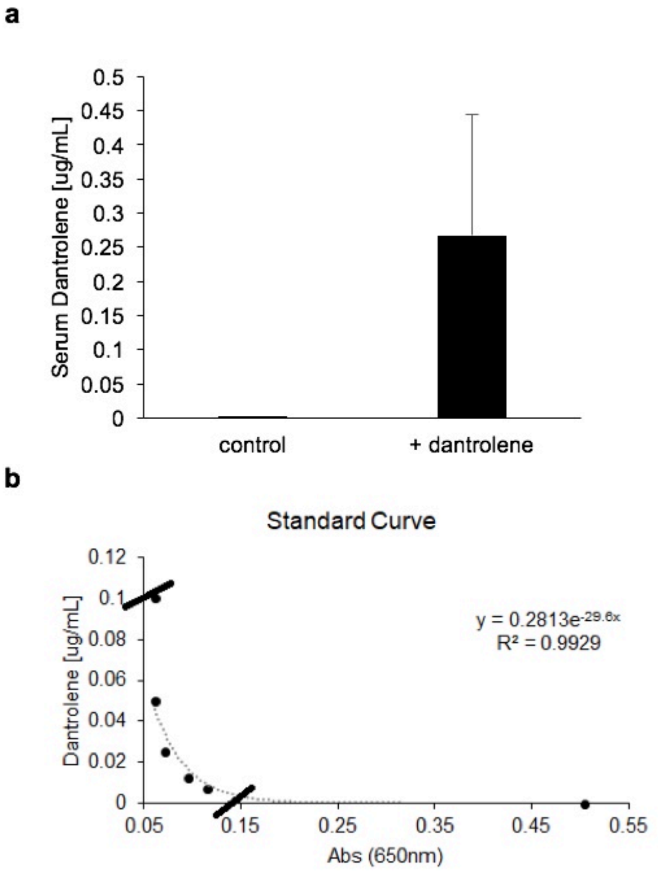

Supplementary Figure 3. Results of Friedman's two-way nonparametric ANOVA on the effect of dantrolene on e23AON treatment for exon 23 skipped / (skipped + unskipped) transcript as measured by ddPCR.

For this analysis, quantitative ddPCR readout of skipped/(skipped+unskipped) for exon-skipping in each mouse was ranked. Once ranked, the findings for each muscle, considered separately, were first fitted for PMO, followed by dantrolene, and the possibility of their interaction.

Friedman's Two-way Nonparametric ANOVA

The ANOVA Procedure

Dependent Variable: RPCR_Q Rank for Variable PCR_Q

Source	DF	Sum of Squares	Mean Square	F Value	Pr > F
Model	7	125825.9029	17975.1290	57.16	<.0001
Error	117	36794.5971	314.4837		
Corrected Total	124	162620.5000			

R-Square	Coeff Var	Root MSE	RPCR_Q Mean
0.773739	28.14871	17.73369	63.00000

Source	DF	Anova SS	Mean Square	F Value	Pr > F
PMO	3	121276.2635	40425.4212	128.55	<.0001
Dantrolene	1	2051.5705	2051.5705	6.52	0.0119
PMO*Dantrolene	3	2498.0689	832.6896	2.65	0.0522

Dependent Variable: RPCRD Rank for Variable PCRD

Source	DF	Sum of Squares	Mean Square	F Value	Pr > F
Model	7	203225.7682	29032.2526	71.56	<.0001
Error	138	55990.2318	405.7263		
Corrected Total	145	259216.0000			

R-Square	Coeff Var	Root MSE	RPCRD Mean
0.784002	27.40496	20.14265	73.50000

Source	DF	Anova SS	Mean Square	F Value	Pr > F
PMO	3	198329.0385	66109.6795	162.94	<.0001
Dantrolene	1	4756.2345	4756.2345	11.72	0.0008
PMO*Dantrolene	3	140.4953	46.8318	0.12	0.9509

Supplementary Figure 4. Results of Friedman's two-way nonparametric ANOVA for the addition of dantrolene to e23AON treatment for dystrophin immunofluorescence quantitation and dystrophin positive fibers.

P-values are from Friedman's test, a two-way nonparametric ANOVA, and meta-analysis of the results across muscles. For this analysis, quantitative dystrophin immunofluorescence quantitation and dystrophin positive fibers.readout in each mouse was ranked.

Friedman's Two-way Nonparametric ANOVA

The ANOVA Procedure

Dependent Variable: RC57_Q Rank for Variable C57_Q

Source	DF	Sum of Squares	Mean Square	F Value	Pr > F
Model	7	229837.8073	32833.9725	69.48	<.0001
Error	145	68523.1927	472.5737		
Corrected Total	152	298361.0000			

R-Square	Coeff Var	Root MSE	RC57_Q Mean
0.770335	28.23216	21.73876	77.00000

Source	DF	Anova SS	Mean Square	F Value	Pr > F
PMO	3	222163.5627	74054.5209	156.70	<.0001
Dantrolene	1	4256.7113	4256.7113	9.01	0.0032
PMO*Dantrolene	3	3417.5333	1139.1778	2.41	0.0693

Dependent Variable: RC57D Rank for Variable C57D

Source	DF	Sum of Squares	Mean Square	F Value	Pr > F
Model	7	300546.6800	42935.2400	188.79	<.0001
Error	151	34341.8200	227.4293		
Corrected Total	158	334888.5000			

R-Square	Coeff Var	Root MSE	RC57D Mean
0.897453	18.85095	15.08076	80.00000

Source	DF	Anova SS	Mean Square	F Value	Pr > F
PMO	3	299112.7209	99704.2403	438.40	<.0001
Dantrolene	1	677.9429	677.9429	2.98	0.0863
PMO*Dantrolene	3	756.0161	252.0054	1.11	0.3478

Dependent Variable: RPostiveQ Rank for Variable PostiveQ

Source	DF	Sum of Squares	Mean Square	F Value	Pr > F
Model	7	246586.4318	35226.6331	81.65	<.0001
Error	147	63418.5682	431.4188		
Corrected Total	154	310005.0000			

R-Square	Coeff Var	Root MSE	RPostiveQ Mean
0.795427	26.62901	20.77062	78.00000

Source	DF	Anova SS	Mean Square	F Value	Pr > F
PMO	3	239888.7374	79962.9125	185.35	<.0001
Dantrolene	1	3234.0959	3234.0959	7.50	0.0069
PMO*Dantrolene	3	3463.5985	1154.5328	2.68	0.0494

Dependent Variable: RPostiveD Rank for Variable PostiveD

Source	DF	Sum of Squares	Mean Square	F Value	Pr > F
Model	7	316449.4676	45207.0668	185.92	<.0001
Error	154	37446.5324	243.1593		
Corrected Total	161	353896.0000			

R-Square	Coeff Var	Root MSE	RPostiveD Mean
0.894188	19.13321	15.59357	81.50000

Source	DF	Anova SS	Mean Square	F Value	Pr > F
PMO	3	312734.6671	104244.8890	428.71	<.0001
Dantrolene	1	2141.8141	2141.8141	8.81	0.0035
PMO*Dantrolene	3	1572.9864	524.3288	2.16	0.0955

Supplementary Figure 5. Results of Friedman's two-way nonparametric ANOVA on the effect of dantrolene on e23AON treatment for dystrophin protein by western blot.

P-values are from Friedman's test, a two-way nonparametric ANOVA, and meta-analysis of the results across muscles. For this analysis, quantitative readout of dystrophin protein by western blot.in each mouse was ranked.

Friedman's Two-way Nonparametric ANOVA

The ANOVA Procedure

Dependent Variable: RWB_Q   Rank for Variable WB_Q

Source	DF	Sum of Squares	Mean Square	F Value	Pr > F
Model	7	135014.2588	19287.7513	42.27	<.0001
Error	124	56582.7412	456.3124		
Corrected Total	131	191597.0000			

R-Square	Coeff Var	Root MSE	RWB_Q Mean
0.704678	32.12251	21.36147	66.50000

Source	DF	Anova SS	Mean Square	F Value	Pr > F
PMO	3	130985.5562	43661.8521	95.68	<.0001
Dantrolene	1	3662.6436	3662.6436	8.03	0.0054
PMO*Dantrolene	3	366.0590	122.0197	0.27	0.8488

Supplementary Figure 6. Combination therapy rescues expression of dystrophin with presence of N-terminal, rod domain, and C-terminal protein sequences.

Dystrophin was detected with antibodies specific to the N-terminal domain (Manex1A), rod domain (MANDYS8), and C-terminal domain (Ab15277) , respectively. ‡"—" defined as 0mg/kg daily dose dantrolene. "+" defined as 30-70mg/kg daily dose dantrolene.

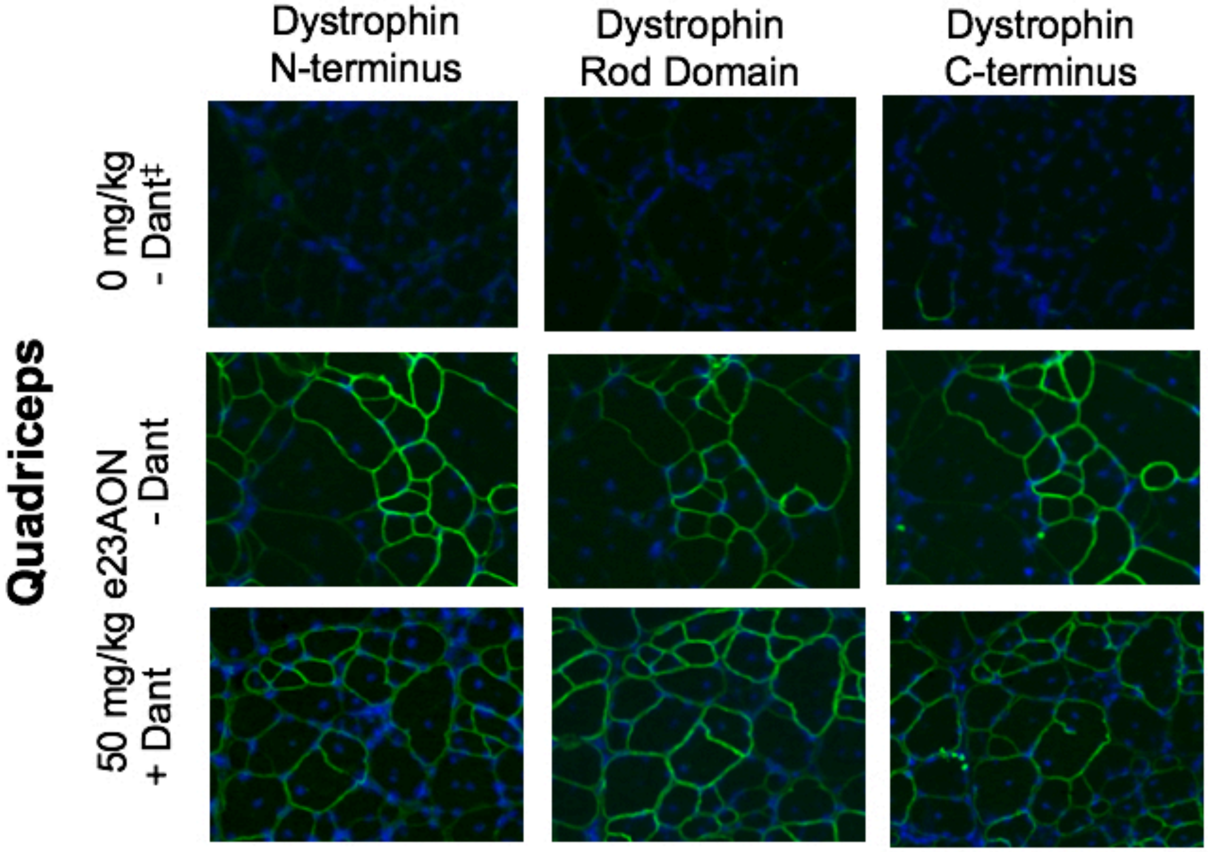

Supplementary Figure 7. Results of Friedman's two-way nonparametric ANOVA on the effect of dantrolene on e23AON treatment for serum CK levels.

P-values are from Friedman's test, a two-way nonparametric ANOVA, and meta-analysis of the results across muscles. For this analysis, quantitative readout of serum CK levels in each mouse was ranked.

Friedman's Two-way Nonparametric ANOVA

The ANOVA Procedure

Dependent Variable: RCK_Serum Rank for Variable CK_Serum

Source	DF	Sum of Squares	Mean Square	F Value	Pr > F
Model	7	76303.8689	10900.5527	11.38	<.0001
Error	125	119736.6311	957.8930		
Corrected Total	132	196040.5000			

R-Square	Coeff Var	Root MSE	RCK_Serum Mean
0.389225	46.19380	30.94985	67.00000

Source	DF	Anova SS	Mean Square	F Value	Pr > F
PMO	3	30801.59067	10267.19689	10.72	<.0001
Dantrolene	1	39072.39425	39072.39425	40.79	<.0001
PMO*Dantrolene	3	6429.88396	2143.29465	2.24	0.0872

Supplementary Figure 8. Results of Friedman's two-way nonparametric ANOVA on the effect of dantrolene on e23AON treatment for centronucleation and eMHC positive fibers. P-values are from Friedman's test, a two-way nonparametric ANOVA, and meta-analysis of the results across muscles. For this analysis, quantitative readout for centronucleation and eMHC positive fibers in each mouse was ranked.

Friedman's Two-way Nonparametric ANOVA

The ANOVA Procedure

Dependent Variable: RCentroQ Rank for Variable CentroQ

Source	DF	Sum of Squares	Mean Square	F Value	Pr > F
Model	7	1501.416667	214.488095	2.02	0.0769
Error	40	4256.083333	106.402083		
Corrected Total	47	5757.500000			

R-Square	Coeff Var	Root MSE	RCentroQ Mean
0.260776	42.10261	10.31514	24.50000

Source	DF	Anova SS	Mean Square	F Value	Pr > F
PMO	3	993.1666667	331.0555556	3.11	0.0369
Dantrolene	1	379.6875000	379.6875000	3.57	0.0662
PMO*Dantrolene	3	128.5625000	42.8541667	0.40	0.7518

Dependent Variable: RCentroD Rank for Variable CentroD

Source	DF	Sum of Squares	Mean Square	F Value	Pr > F
Model	7	4773.000000	681.857143	7.82	<.0001
Error	40	3489.000000	87.225000		
Corrected Total	47	8262.000000			

R-Square	Coeff Var	Root MSE	RCentroD Mean
0.577705	38.12013	9.339433	24.50000

Source	DF	Anova SS	Mean Square	F Value	Pr > F
PMO	3	3973.500000	1324.500000	15.18	<.0001
Dantrolene	1	546.750000	546.750000	6.27	0.0165
PMO*Dantrolene	3	252.750000	84.250000	0.97	0.4183

Dependent Variable: ReMHCQ Rank for Variable eMHCQ

Source	DF	Sum of Squares	Mean Square	F Value	Pr > F
Model	7	6486.250000	926.607143	13.62	<.0001
Error	40	2722.250000	68.056250		
Corrected Total	47	9208.500000			

R-Square	Coeff Var	Root MSE	ReMHCQ Mean
0.704376	33.67192	8.249621	24.50000

Source	DF	Anova SS	Mean Square	F Value	Pr > F
PMO	3	5587.375000	1862.458333	27.37	<.0001
Dantrolene	1	391.020833	391.020833	5.75	0.0213
PMO*Dantrolene	3	507.854167	169.284722	2.49	0.0743

Dependent Variable: ReMHCD Rank for Variable eMHCD

Source	DF	Sum of Squares	Mean Square	F Value	Pr > F
Model	7	6192.946429	884.706633	17.67	<.0001
Error	38	1902.553571	50.067199		
Corrected Total	45	8095.500000			

R-Square	Coeff Var	Root MSE	ReMHCD Mean
0.764986	30.10986	7.075818	23.50000

Source	DF	Anova SS	Mean Square	F Value	Pr > F
PMO	3	5742.540559	1914.180186	38.23	<.0001
Dantrolene	1	272.695652	272.695652	5.45	0.0250
PMO*Dantrolene	3	177.710217	59.236739	1.18	0.3290
